# Assembly and Covalent Cross-Linking of an Amine-Functionalised Metal-Organic Cage

**DOI:** 10.3389/fchem.2021.696081

**Published:** 2021-05-25

**Authors:** Matthew L. Schneider, Adrian W. Markwell-Heys, Oliver M. Linder-Patton, Witold M. Bloch

**Affiliations:** Department of Chemistry, The University of Adelaide, Adelaide, SA, Australia

**Keywords:** cage compound, self-assembly, coordination, cross-linking, amine-functionality, porosity

## Abstract

The incorporation of reactive functional groups onto the exterior of metal-organic cages (MOCs) opens up new opportunities to link their well-defined scaffolds into functional porous solids. Amine moieties offer access to a rich catalogue of covalent chemistry; however, they also tend to coordinate undesirably and interfere with MOC formation, particular in the case of Cu_2_ paddlewheel-based MOCs. We demonstrate that tuning the basicity of an aniline-functionalized ligand enables the self-assembly of a soluble, amine-functionalized Cu_4_L_4_ lantern cage (**1**). Importantly, we show control over the coordinative propensity of the exterior amine of the ligand, which enables us to isolate a crystalline, two-dimensional metal-organic framework composed entirely of MOC units (**2**). Furthermore, we show that the nucleophilicity of the exterior amine of **1** can be accessed in solution to generate a cross-linked cage polymer (**3**) via imine condensation.

## Introduction

The applications of metal-organic frameworks (MOFs) have been rapidly expanding, with recent studies utilizing MOFs as crystallization matrices (Bloch et al., 2015), sacrificial scaffolds for hierarchical porosity (Dang et al., 2017; Haase et al., 2020), and protective agents in biomedicine (Yang and Yang, 2020; Liang et al., 2021). The attractive properties of MOFs have largely been underpinned by important design principles surrounding their net topology (Robson 2000), isoreticular chemistry (Furukawa et al., 2013), and post-synthetic chemistry (Mandal et al., 2021). Over the past decade, it has been demonstrated that discrete molecular compounds, such as porous organic cages and metal-organic cages (MOCs), possess many of the attractive properties of MOFs (Hasell and Cooper, 2016; Lee et al., 2021; Pullen and Clever, 2018). Unlike MOFs, MOCs are inherently soluble, and this characteristic opens up avenues for solution processing and hybridization with organic polymers (Pastore and Cook, 2020). In this context, a particularly accessible class of MOCs, based on di-carboxylate ligands and M_2_ paddlewheel nodes (M = Cu^2+^, Rh^2+^ Mo^2+^ and Cr^2+^), has received significant scientific attention. Recent studies have demonstrated several impressive properties for MOCs based on M_2_ paddlewheel nodes, including tunable porosity (Gosselin et al., 2020), solvatomorphism (Craig et al., 2018; Bloch et al., 2020), hydrolytic stability (Lal et al., 2019a) (Mollick et al., 2019), and transport capabilities (Grancha et al., 2019).

More recently, approaches to directly polymerize soluble MOCs into polymers with intrinsic microporosity have been developed (El-Sayed et al., 2019). These kinds of materials can possess porosity without the need for long-rage order, and improved mechanical properties over MOFs. (Lal et al., 2019b; Zhao et al., 2020; Carné-Sánchez et al.,; Schneider et al., 2020; Lal et al., 2019a; Shao et al., 2020). Advances in this area have been made possible by fundamental studies into the compatibility of M_2_ paddlewheel-based MOCs with various surface functionalities. These include coordinatively competitive groups such as hydroxyl (Niu et al., 2015), carboxylate (Albalad et al., 2019), carbonyl (Bloch et al., 2020), and amine substituents (Albalad et al., 2019; Schneider et al., 2020; Taggart et al., 2020) which can be installed onto the MOC exterior through ligand design or post-assembly modifications (Zeng et al., 2020). Of these functionalities, amine moieties offer considerable synthetic versatility for post-assembly reactions, owing to their tunable basicity and nucleophilicity. We note that MOCs based on mononuclear metal nodes or Zr-O metal clusters can be readily assembled in the presence of amine groups (Wang et al., 2011; Nam et al., 2017; Roberts et al., 2018). This may relate to the inertness of the metal centre and propensity to form stable complexes with chelating ligands in favour of monodentate amine donors (Roberts et al., 2014). For MOCs assembled from M_2_ paddlewheel nodes however, the axial coordination sites are inevitably occupied by weakly coordinating solvent which can be readily displaced by an amine donor. Thus, incorporating amine functionality onto the surface of MOCs composed of paddlewheel nodes presents a significant challenge (Chen et al., 2016).

**FIGURE 1:**
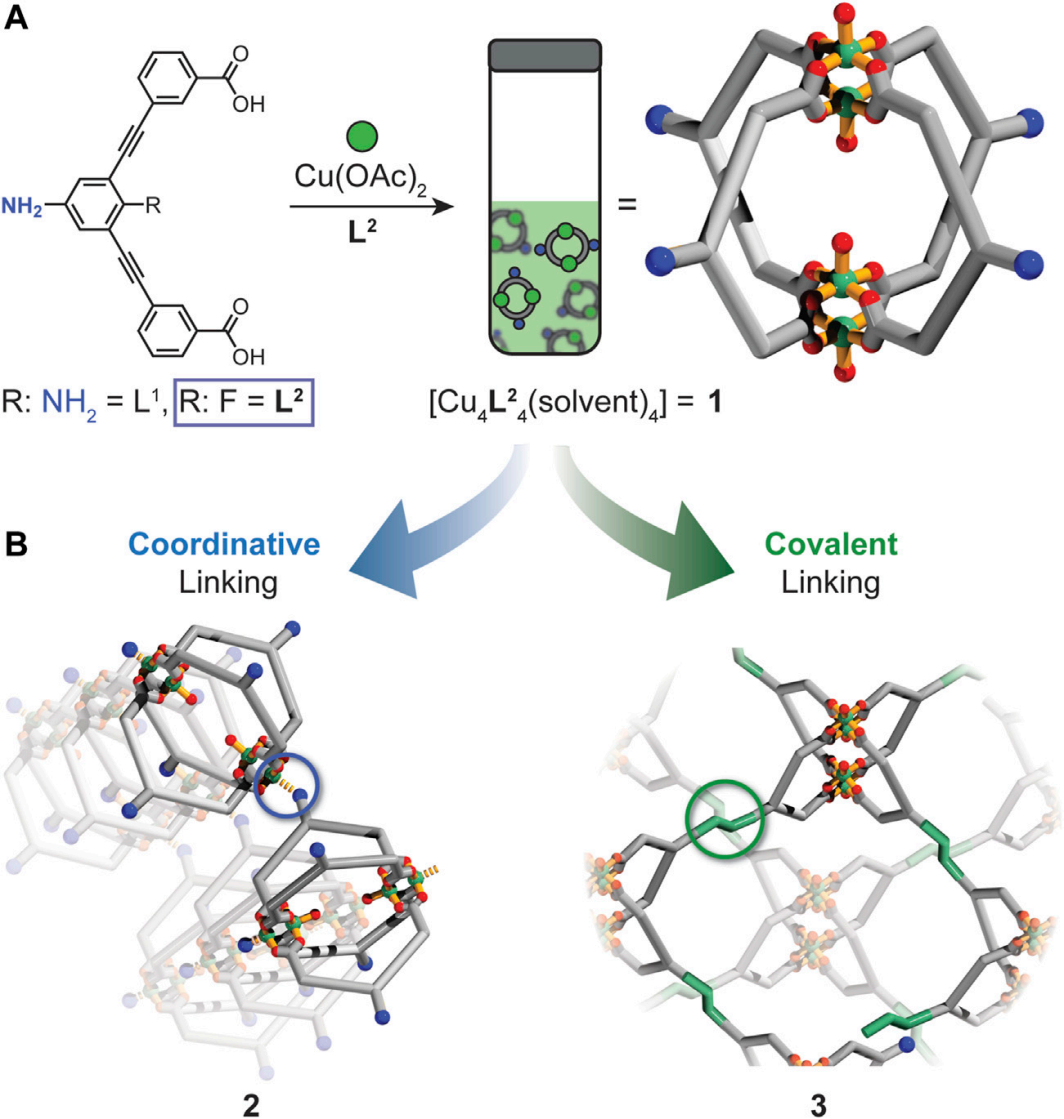
**(A)** Solution self-assembly of **1** from **L** ^**2**^ and Cu(OAc)_2_ in DMF or DMA. **(B)** The exterior amine of **1** can behave as a donor or nucleophile to link **1** into two different types of metal-organic cage polymers (**2** or **3**).

Herein we report the self-assembly and covalent cross-linking of a highly versatile Cu_4_L_4_ MOC (**1**) with pendant amine functionality (Figure 1A). By tuning the basicity of the amine moiety of the ligand, we show that the exterior amine substituents of **1** participate in aniline-copper coordination only upon crystallization (Figure 1B). This facilitates the formation of a crystalline two-dimensional MOF where **1** serves as a four-connecting node. Furthermore, we demonstrate that the amine moieties of **1** can be accessed for imine condensation, enabling the formation of a covalently cross-linked MOC polymer. Using a combination of solution and solid-state techniques, we characterize the structures of the cage-based polymers **2** and **3** and assess their porosity by adsorption experiments. Thus, the work described herein sheds light on the chemistry of amine-functionalized MOCs and their utilization in the synthesis of cage-based polymeric solids (Figure 1).

## Results and Discussion

### Ligand Design and Synthesis

In our previous work, we demonstrated that **L** ^**1**^ (Figure 1A)—a dicarboxylate ligand with a phenylene diamine core—undergoes spontaneous polymerization in the presence of Cu(OAc)_2_ via copper-aniline coordination. The Cu_4_L_4_ cage could only be accessed by covalently masking the exterior amine of **L** ^**1**^ using Boc or Fmoc protection chemistry. However, upon unmasking the amine functionality in the assembled MOC, an amorphous coordination polymer was obtained (Schneider et al., 2020). For the present work, we rationalized that reducing the ligand’s aniline basicity should render it coordinatively inactive, thus facilitating MOC assembly in solution. Consequently, we devised a synthetic route to a dicarboxylate ligand with 4-fluoroaniline backbone (Figure 1A, **L** ^**2**^). **L** ^**2**^ includes an exterior aniline with a pKa = 4.6, which is significantly lower than the phenylene diamine core of **L** ^**1**^ (pKa = 6.2) (Perrin, 1965). The synthesis of **L** ^**2**^ began with a Sonogashira cross-coupling between 2-fluoro-1,3-diiodo-5-nitrobenzene (**4**) and methyl 3-ethynylbenzoate (**5**). Subsequent reduction of the pendant nitro group with tin(II) chloride, followed by ester hydrolysis yielded 3,3′-((5-amino-2-fluoro-1,3-phenylene)bis(ethyne-2,1-diyl))dibenzoic acid, **L** ^**2**^.

### Assembly and Characterization of MOC 1 and MOF 2

**FIGURE 2:**
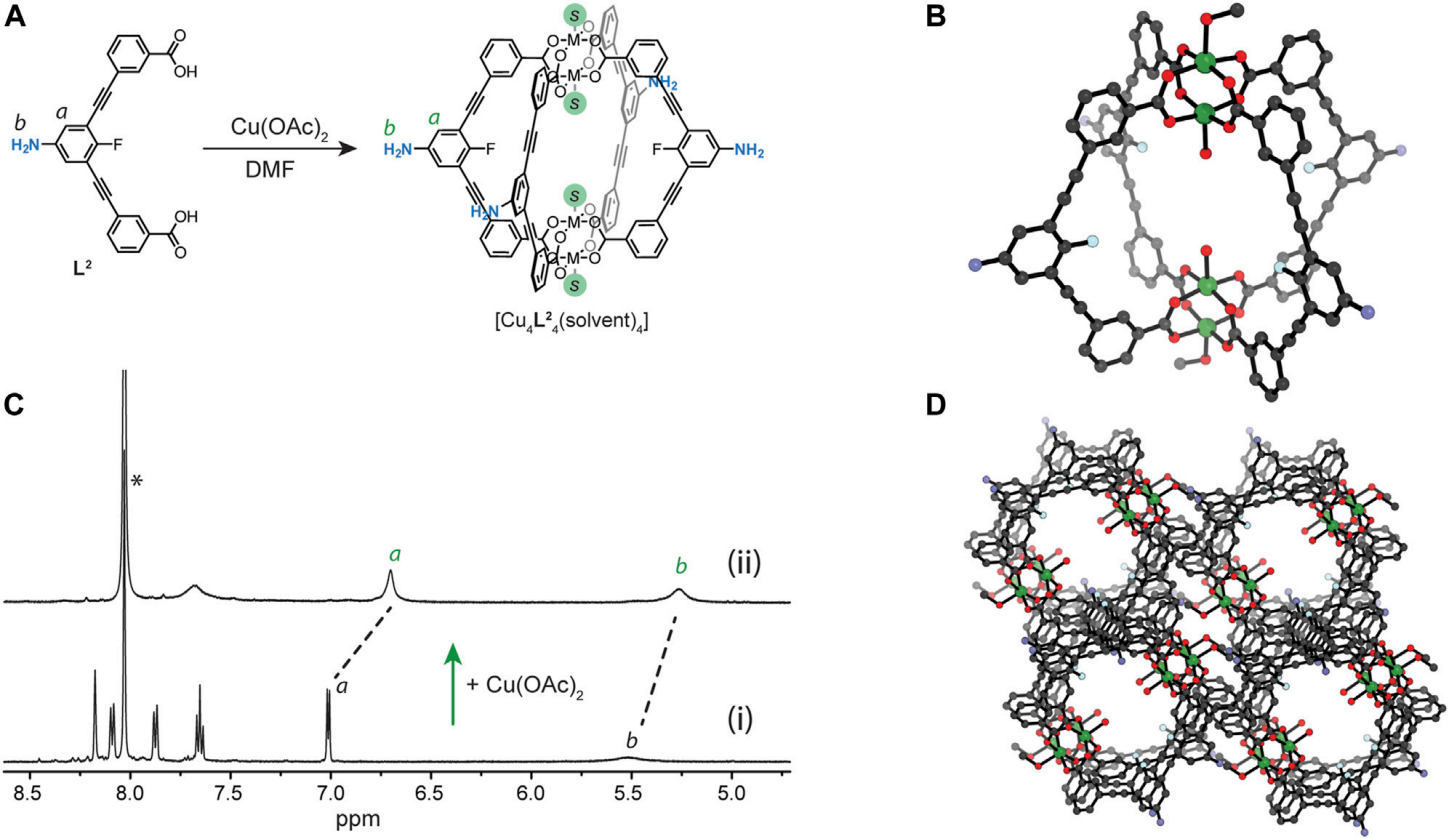
**(A)** A scheme showing the Cu(II)-mediated self-assembly of Cu_4_ **L** ^**2**^_4_ (**1**); **(B)** ^1^H NMR spectrum (500 MHz/DMF-d_7_) showing MOC assembly in solution: (i) **L** ^**2**^ and (ii) **L** ^**2**^ after the addition of 1.1 equivalents of Cu(OAc)_2_; **(C)** SCXRD of $\left[{\text{Cu}}_{4}L_{4}^{2}{\left(\text{MeOH}\right)}_{2}{\left({\text{H}}_{2}\text{O}\right)}_{2}\right]$ (**1**); **(D)** crystal-packing of **1** along the *b* axis.

Combining equimolar ratios of **L** ^**2**^ and Cu(OAc)_2_ in DMF produced a transparent green solution of **1** (Figure 2A). In the ^1^H NMR spectrum (DMF-d_7_), the proton signals of **L** ^**2**^ were completely consumed within 5 min and the key resonances of the paramagnetic cage **1** (including the broad resonances of backbone proton *b* and amine resonance *a*, Figure 2B) were observed upfield shifted relative to the free ligand. Further evidence for the formation of **1** was obtained from the UV-Vis spectrum, which showed the characteristic Cu_2_(COO)_4_ band at 728 nm (Supplementary Figure S5). Crystals suitable for single-crystal X-ray diffraction (SCXRD) were obtained from a 2.2:1 MeOH/DMA solution of **1**. **1** crystallises in the triclinic space group *P*1 with half of the ${\text{Cu}}_{4}L_{4}^{2}$ cage in the asymmetric unit. Each Cu_2_ paddlewheel is coordinated by four carboxylate donors with MeOH and H_2_O ligands occupying the exterior and interior axial sites, respectively (Figure 2C). The pendant amine substituent of the ligand participates in hydrogen bonding with an oxygen from a carboxylate donor of an adjacent cage (average D_NH···O_ = 2.25 Å, angle = 158.1°). In addition, the aniline and carboxylate moieties of the ligand participate in antiparallel face-to-face π-stacking between adjacent cage molecules (average distance = 3.24 Å). Open pores intrinsic to the cage are apparent along the crystallographic *b* axis (∼6 × 8 Å) due to the eclipsed arrangement of the MOC structure (Figure 2D).

**FIGURE 3:**
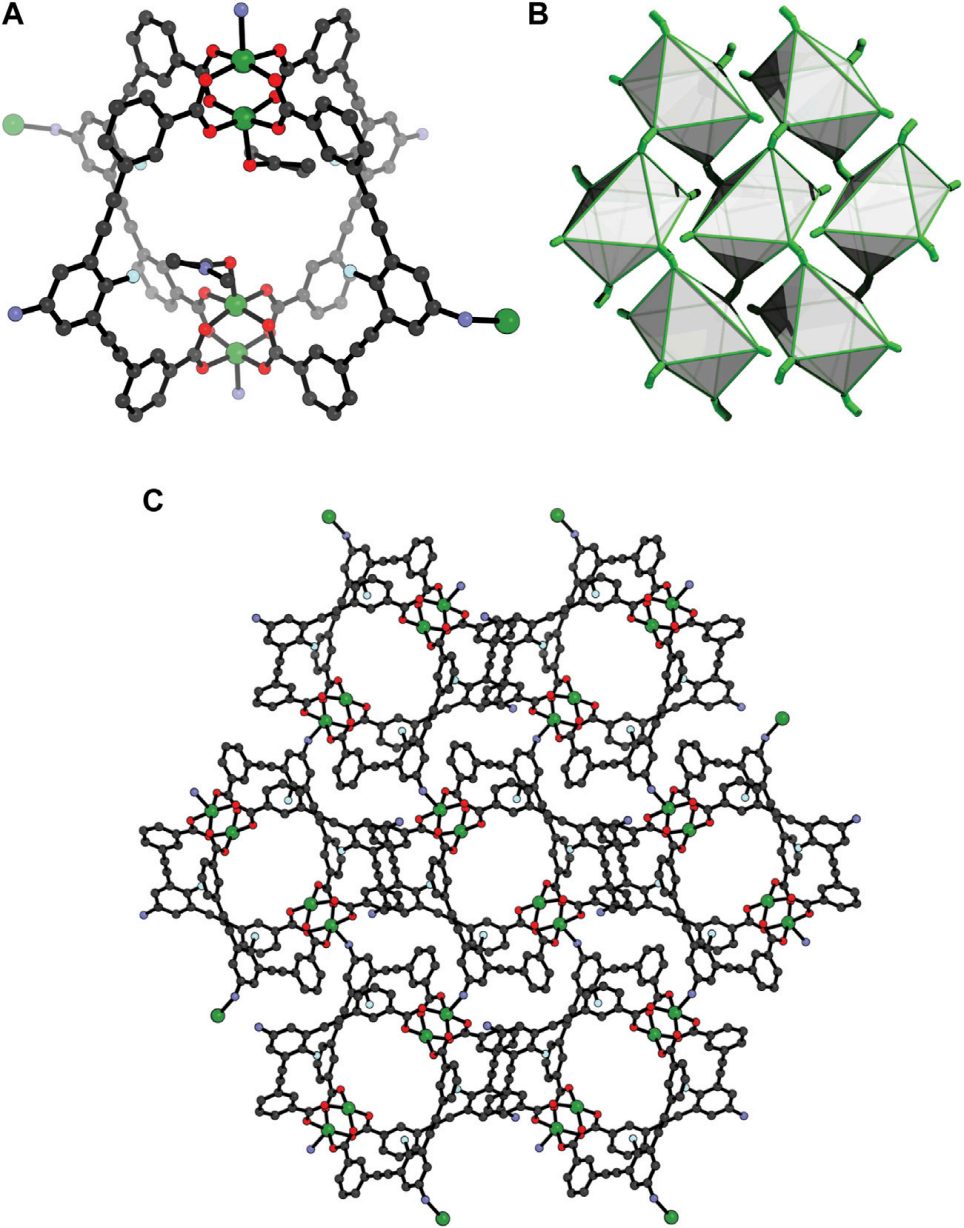
X-ray structure of **2 (A)** the Cu_4_ **L** ^**2**^_4_ unit showing the coordination to Cu(II) by two ligands in the *trans* direction; **(B)** The 4,4 net of **2**, with each cage represented as a polyhedron; **(C)** crystal-packing of **2** along the *a* axis (coordinated and pore-bound solvent removed for clarity).

Owing to solubility of **1** in DMF and DMA, we explored whether other crystallisation conditions can lead to a different crystal-packing through solvatomorphism, (Bloch et al., 2020; Deegan et al., 2021). Indeed, slow vapour diffusion of diisopropyl ether (DiPE) into a DMA solution of the cage yielded crystals of **2** after 4 days. SCXRD revealed that **2** crystallises in the monoclinic space group *P*2_1_/*c* with half of a ${\text{Cu}}_{4}L_{4}^{2}$ cage moiety in the asymmetric unit. Each Cu_2_ paddlewheel is coordinated by four carboxylate donors of **L** ^**2**^ and a DMA ligand, the latter occupying the interior axial site (Figure 3A). Interestingly, both exterior Cu_2_ axial sites are coordinated by an amine donor of an adjacent molecule of **1**, thereby forming a two-dimensional metal-organic framework. Since two exterior Cu_2_ axial sites of each cage are available for coordination, only two ligands from each cage link to adjacent cage molecules via their peripheral amine donors (D_Cu···N_ = 2.19 Å, Cu-N-C angle = 111.9°). The nitrogen of the non-coordinating amine of the Cu_4_L_4_ unit accepts a hydrogen bond from a coordinating amine substituent of an adjacent cage molecule within the same layer (D_NH···N_ = 2.18 Å, angle = 153.0°). Considering that each MOC is coordinated by two other MOCs (via the axial Cu_2_ coordination sites) and also coordinates to two separate MOCs in the *trans* direction, the ${\text{Cu}}_{4}L_{4}^{2}$ unit acts as a four-connecting node (Figures 3B,C). This connectivity gives rise to a 2D MOF with an eclipsed 4,4 net (Figure 3).

Characterisation of the bulk sample of **2** was carried out by Powder X-ray Diffraction (PXRD) and Thermogravimetric Analysis (TGA). The latter revealed that **2** is thermally stable up to ∼280°C (Supplementary Figure S15), which is typical for Cu_2_-paddlewheel MOCs. A Rietveld refinement of the PXRD data yielded a goodness-of-fit (GoF) of 1.96 and a R_p_ of 5.79, indicating that the bulk crystalline sample is in good agreement with the single-crystal X-ray data (Supplementary Figure S13). The reason behind the bulk crystallisation of **2** over the discrete cage **1** may relate to the propensity of the respective solvent to compete with the amine donors for coordination to the available Cu_2_ axial sites. MeOH is considered a marginally stronger ligand than DMA (Díaz-Torres and Alvarez, 2011), which may explain why in the presence of MeOH, the discrete MOC is crystallised, rather than the 2D MOF. Interestingly, sonication of crystals of **2** in DMF-d_7_ resulted in complete dissolution, and analysis by ^1^H NMR spectroscopy revealed identical proton resonances to that of the discrete MOC **1** (Supplementary Figure S2). Therefore, the aniline-copper coordination bonds that propagate the framework of **2** are relatively weak and can be readily displaced by solvent.

### Post-Assembly Covalent Cross-Linking of 1

**FIGURE 4:**
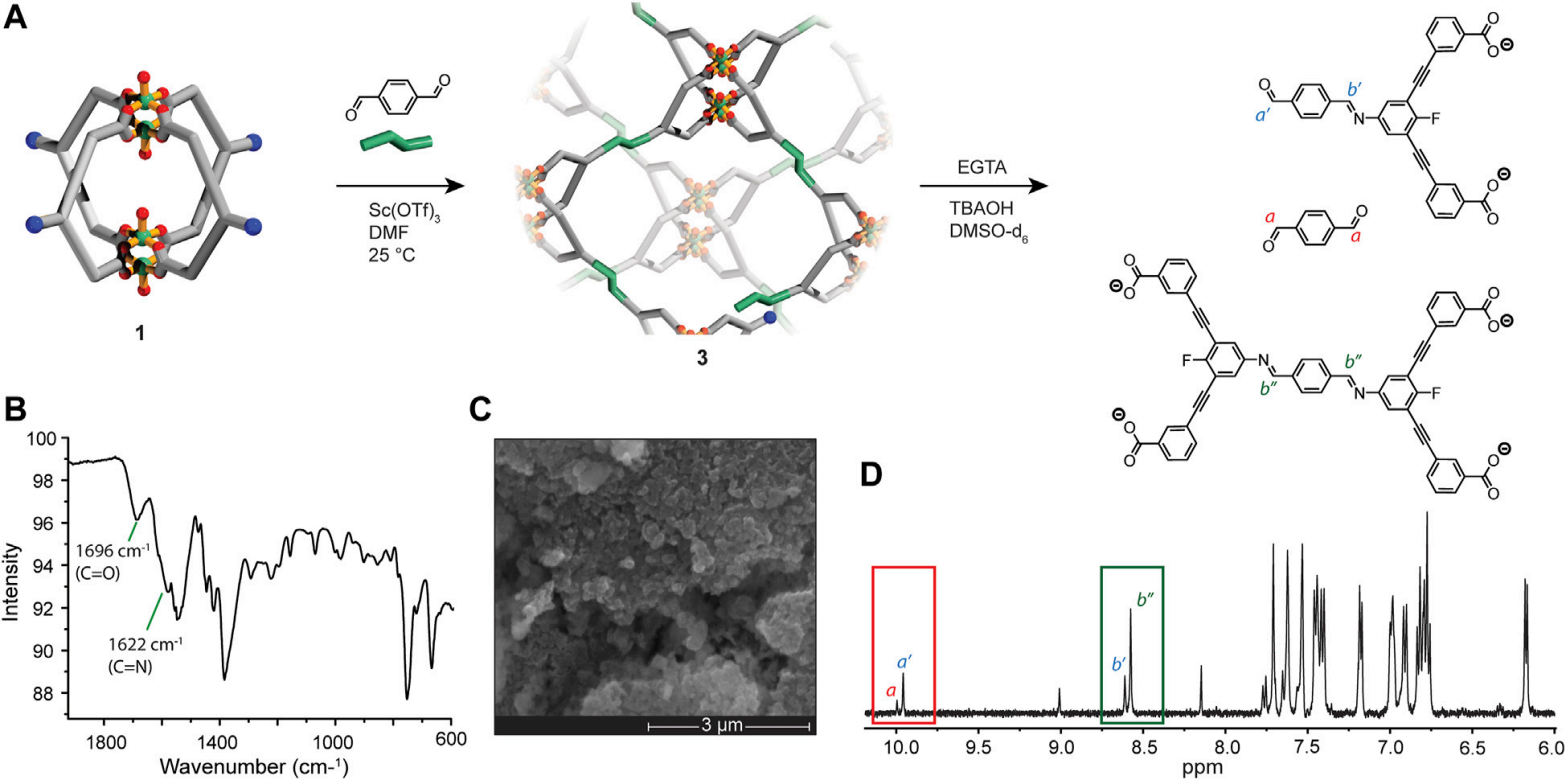
**(A**) A scheme showing the covalent-cross linking of **1** (in solution) with PDA to form **3**; **(B**) IR spectrum of **3**, showing C=O (from carboxylate and carbonyl moieties) and C=N (imine bond) stretches; **(C)** SEM image of **3**, 50 K magnification; **(D)** ^1^H NMR spectrum (500 MHz, DMSO-d_6_) of a digested sample of **3**, with disassembled fragments shown above and key resonances annotated on the spectrum [unreacted **L** ^**2**^ is not shown in **(A)**].

The solubility of **1** led us to investigate whether the nucleophilicity of the exterior amine groups can be accessed for covalent cross-linking. Considering that the four amine groups of **1** lie along the same plane, we rationalised that a condensation with a linear molecule such as terephthaldehyde (PDA) may give rise to a 2D network if a suitable level of reversibility is achieved(Lin et al., 2015). Common reaction conditions used in the synthesis of covalent-organic frameworks (COFs) typically utilise high temperatures and employ acetic acid as the catalyst (Li et al., 2020). These conditions, however, are not compatible with the labile Cu_2_-carboxylate bonds that comprise the Cu_4_L_4_ MOC. In our previous work, we found that post-assembly imine condensations can be carried out on Cu_4_L_4_ MOCs using a Sc(OTf)_3_ catalyst under mild conditions. (Bloch et al., 2020; Matsumoto et al., 2017). Thus, we employed the same conditions for the covalent cross-linking of **1**. Upon combing **1** and PDA in DMA in a 0.4:1 ratio (with respect to **L** ^**2**^), a clear green solution was obtained. After the addition of 0.04 equivalents of Sc(OTf)_3_ and allowing the reaction mixture to stand at 25°C for 8 h, an amorphous precipitate (**3**) was obtained. In order to remove traces of Sc(OTf)_3_ and unreacted PDA, the solid was thoroughly washed with DMSO as well as MeOH. SEM/EDX analysis revealed that **3** contains copper co-localised with carbon, nitrogen and oxygen in monodispersed (<50 nm) particles (Figure 4C; Supplementary Figure S24). Infrared spectroscopy (IR) revealed stretches at 1,696 cm^−1^ and 1,622 cm^−2^; the former corresponding to the carboxylate groups of the ligand and unreacted carbonyl moieties, and the latter to newly formed imine bonds of the network (Figure 4B). To assess the efficacy of the MOC cross-linking, we digested a sample of **3** using a protocol based on Ethylene glycol-bis(2-aminoethylether)-N,N,N′,N′-tetraacetic acid (EGTA). This digestion method results in metal-ligand dissociation without perturbing imine bonds (Markwell-Heys et al., 2021). 1D and 2D H NMR analysis of the digested sample revealed the presence of unreacted **L** ^**2**^, a mono-imine linked adduct, and a di-imine linked adduct in a ratio of 6:1:3, respectively (Figures 4A, D; Supplementary Figure S3). Based on this ratio, a MOC cross-linking efficacy of 30% was achieved, suggesting non-continuity in the polymer structure and the possibility of unlinked fragments trapped within the solid.

### Adsorption Analysis of MOF 2 and Cross-Linked Polymer 3

**FIGURE 5:**
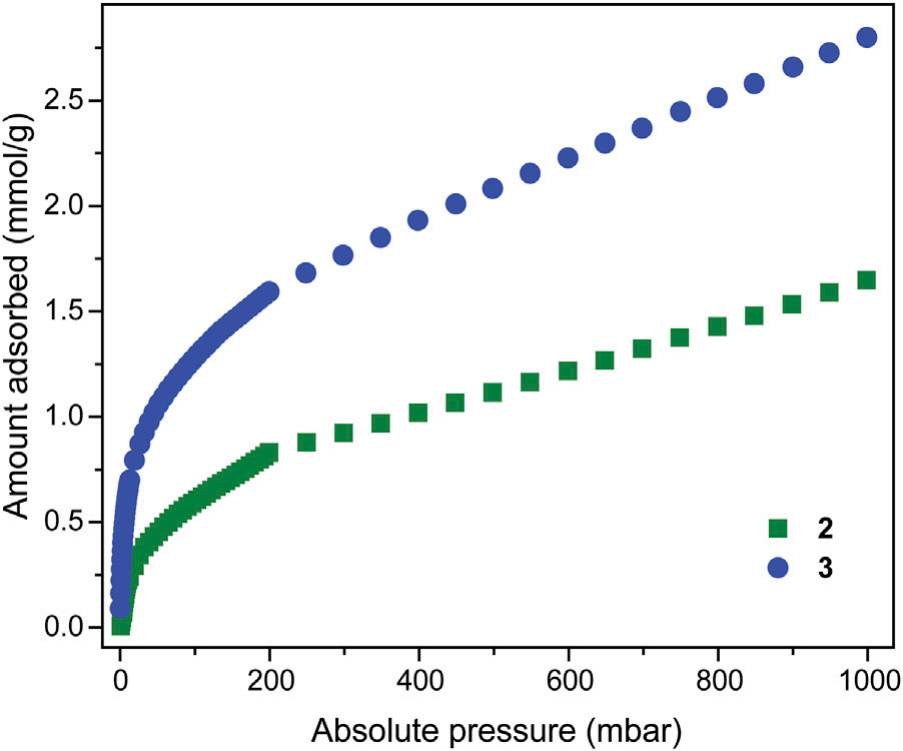
195 K CO_2_ isotherm of MOF **2** (green squares) and cross-linked polymer **3** (blue circles).

In order to investigate the porosity of **2** and **3**, we subjected the solvent-exchanged solids to high vacuum at 90°C for 6 h (supporting information). PXRD analysis of the activated form of **2** revealed a complete loss of long-range order (Supplementary Figure S9). This is not surprising given the weak aniline-copper interaction responsible for propagating the 2D network, and the dependence on lattice-bound solvent for solid-state organisation. Measurement of the 77 K N_2_ adsorption isotherms revealed that **2** is essentially non-porous, whilst **3** possesses a type-I/II N_2_ adsorption isotherm, with a sharp uptake in the low-pressure region, followed by a gradual increase due to multi-layer formation on the external surface of the solid particles (Supplementary Figure S18). The 195 K CO_2_ isotherms of **2** and **3**, however, revealed that both solids are both microporous and possess a type I profile, with a maximum uptake of 1.65 mmol/g and 2.8 mmol/g at 1 bar, respectively (Figure 5). Thus, the BET surface areas derived from these isotherms are 51 and 97 m^2^g^−1^ for **2** and **3** respectively. For **2**, the loss of long-range order that occurs upon activation may be associated with irregular movement of the 2D layers, thus blocking the available pores and reducing the accessible surface area. For the cross-linked polymer **3**, the largely amorphous structure is consistent with a variable degree of covalent links between MOC nodes. Given the degree of cross-linking (30%), it is likely that significant defects are present within structure, which may also lead to trapping of residual unreacted PDA. Together, these factors contribute to a lower porosity for **3** than anticipated. Interestingly, activation of the discrete MOC solid **1** was not associated with a complete loss of crystallinity (Supplementary Figure S10). **1** was found to be completely non-porous to N_2_, but porous to CO_2_ at 195 K (Supplementary Figures S19, S21), with a derived BET surface area of 95 m^2^g^−1^. These experiments highlight the difficulty in predicting porosity for amorphous materials, particularly when structural rearrangements accompany their activation. Indeed, approaches to stabilise MOC solids and mitigate their structural collapses are currently being developed (Mollick et al., 2019).

## Conclusion

In summary we have reported the synthesis, crystallisation and post-assembly cross-linking of a soluble, amine-functionalised Cu_4_L_4_ MOC (**1**). **1** could be isolated in its discrete form both in solution and the solid-state. Crystallisation of **1** from weakly-coordinating solvent facilitated aniline-copper coordination to produce a crystalline 2D MOF. In this structure, the ${\text{Cu}}_{4}L_{4}^{2}$ unit behaved as a four-connecting node by virtue of two coordinating amine donors and two available axial coordination sites per cage. Owing to the solubility of **1,** we demonstrated that the amine moieties of the MOC can be utilised for imine condensation to produce a covalently cross-linked polymer with an amorphous structure. A digestion protocol used to directly elucidate the degree of cross-linking revealed an overall efficacy of 30%. CO_2_ adsorption measurements at 195 K revealed a porosity of <100 m^2^g^−1^ for MOC polymers **2** and **3**, and therefore, optimisation of the solvent exchange and activation protocols are a future goal. This work highlights the importance of ligand design in promoting self-assembly of a soluble, amine-functionalised Cu_4_L_4_ MOC. The successful covalent cross-linking of **1** opens up new avenues for fine-tuning the porosity by controlling the degree of cross-linking through modulating reagent stoichiometry and reaction conditions. We envision that this chemistry will lay a foundation for the development of a range of MOC-derived porous solids and current work in our laboratory is focused in this direction.

## Experimental

### Materials and Characterisation

Unless otherwise stated, all chemicals were obtained from commercial sources and used as received. Compounds **4** and **5** were synthesised according to literature procedures (Blake et al., 2020; Bloch et al., 2020). Triethyl amine (TEA) was distilled from KOH and stored under argon. Tetrahydrofuran (THF) was freshly distilled using a sodium/benzophenone solvent still. Electrospray ionization (ESI) mass spectra were recorded on an Agilent 6230 TOF LCMS. Infrared spectra were collected on a Perkin-Elmer Spectrum 100 using a UATR sampling accessory. Thermal gravimetric analysis (TGA) was performed on a F3 Jupiter STA + QMS instrument under a constant flow of N_2_ 80% and O_2_ 20% at a temperature increase rate of 10°C/min. High field NMR spectra were recorded using an Agilent 500 MHz spectrometer. Whilst the anomalous paramagnetism of the Cu_2_ paddle-wheel precluded a complete assignment of proton resonances of **1**, resonances not immediately adjacent to the metal nodes were clearly resolved and could be assigned (Brega et al., 2015; Bloch et al., 2020). Powder X-ray diffraction data were collected on a Bruker Advanced D8 diffractometer (capillary stage) using Cu Kα radiation (λ = 1.5418 Å, 50 kW/40 mA) Simulated powder X-ray diffraction patterns were generated from the single crystal data using Mercury 4.3.1 Gas sorption isotherm measurements were performed on a Micromeritics 3Flex Surface Characterisation Analyser. UHP grade (99.999%) N_2_ was used for all measurements. Temperatures were maintained at 77 K using a cryo-cooler. The isotherms were then analysed to determine the Brunauer Emmet-Teller (BET) surface area and pore-size distribution using the MicroActive software (Version 3.00, Micromeritics Instrument Corp. 2013).

### Synthesis of 6

**4** (0.51 g, 1.30 mmol), **5** (0.75 g, 4.68 mmol), THF (25 ml), and diisopropylamine (6 ml) were added to a Schlenk flask and the resulting mixture was degassed with Ar for 30 min. Pd(PPh_3_)_2_Cl_2_ (100 mg) was added in one portion, and the reaction mixture was heated at 40°C for 48 h. The reaction mixture was filtered and the precipitate was washed with EtOAc (2 × 20 ml). The filtrate was reduced *in vacuo* to give a black crude gum, which was diluted with Et_2_O (100 ml) and filtered. The crude precipitate was washed with Et_2_O (2 × 50 ml) and was purified via flash column chromatography (CH_2_Cl_2_) to afford **6** as a pale-yellow solid (0.49 g, 82%). ^1^H NMR (500 MHz, CDCl_3_) δ 8.35 (2 H, d, J = 5.7 Hz), 8.25 (2 H, t, J = 1.8 Hz), 8.06 (2 H, dd, J = 7.8, 1.5 Hz), 7.76 (2 H, dd, J = 7.8, 1.5 Hz), 7.48 (2 H, t, J = 7.9 Hz), 3.94 (6 H, s). ^13^C NMR (126 MHz, CDCl_3_) δ 169.29, 168.73, 167.17, 146.34, 138.58, 135.70, 133.40, 133.14, 131.43, 130.86, 124.75, 116.40, 116.26, 99.09, 83.37, 79.96, 79.70, 79.45, 55.05, 49.00, 11.29. *ν*_max_ (neat, cm^−1^): 1713 (s, HC = O), 1,538 (m), 1,438 (m), 1,436 (m), 1,387 (m), 1,362 (m, O=N=O). ESI-MS (C_26_H_16_F_1_N_1_O_6_): calc: 457.0962 [M + H]^+^; found 457.1012.

### Synthesis of 7

**6** (0.48 g, 1.05 mmol) was added in one portion to a suspension of SnCl_2_.HCl (1.00 g, 4.43 mmol) in EtOH (80 ml) at room temperature. The reaction mixture was heated at 50°C for 16 h, before being allowed to cool to room temperature. The reaction mixture was filtered through celite and the solvent was reduced *in vacuo* till approximately 10 ml remained, before being diluted with water (100 ml) and toluene (4 × 50 ml). The organics were combined, washed with water (50 ml), brine (50 ml), dried over MgSO_4_ and reduced *in vacuo* to afford **7** as a solid (0.32 g, 71%). ^1^H NMR (500 MHz, CDCl_3_) δ 8.22 (2H, t, J = 1.8 Hz), 8.02 (2H, dd, J = 7.8, 1.5 Hz), 7.72 (2 H, dd, J = 7.8, 1.5 Hz), 7.45 (2H, t, J = 7.9 Hz), 6.81 (2 H, d, J = 5.7 Hz), 3.94 (6H, s), 3.63 (2H, s). ^13^C NMR (126 MHz, CDCl_3_) δ 168.99, 139.17, 138.46, 136.32, 135.51, 133.19, 132.28, 131.31, 131.20, 125.90, 122.00, 95.95, 85.97, 79.90, 79.65, 79.40, 54.95. *ν*_max_ (neat, cm^−1^): 3,460 (w), 3,378 (w, NH_2_), 1722 (s, HC = O), 1,594 (C=C), 1,439 (m), 1,436 (m), 1,294 (m). ESI-MS (C_26_H_18_F_1_N_1_O_4_): calc: 428.1293 [M + H]^+^; found 428.1283.

### Synthesis of L^2^

KOH (0.06 g, 1.03 mmol) in water (5 ml) was slowly added dropwise (over 30 min) to a solution of **7** (0.20 g, 0.47 mmol) in THF (20 ml) at room temperature. The reaction mixture was stirred at room temperature for 2 h, and the solvent was reduced to 5 ml under a stream of N_2_. The residue was acidified to pH 4 with 3M HCl and the resulting precipitate was isolated under reduced pressure, followed by washing with water and dried under reduced pressure to afford **L** ^**2**^ as a light brown solid (0.12 g, 65%). ^1^H NMR (500 MHz, DMSO) δ 13.18 (2H, s), 8.02 (2H, t, J = 1.8 Hz), 7.97 (2H, dd, J = 7.8, 1.5 Hz), 7.79 (2H, dd, J = 7.8, 1.5 Hz), 7.57 (2H, t, J = 7.9 Hz), 6.81 (2H, d, J = 5.7 Hz), 5.34 (2H, s). ^13^C NMR (126 MHz, DMSO) δ 169.54, 147.70, 138.53, 135.09, 134.60, 132.96, 132.50, 125.26, 121.53, 113.92, 113.79, 95.84, 86.41. *ν*_max_ (neat, cm^−1^): 2,970 (br, w) 1,693 (s, HC = O), 1,585 (m, C=C), 1,450 (m), 1,391 (m), 1,362 (m). ESI-MS (C_24_H_14_F_1_N_1_O_4_): calc: 400.0980 [M + H]^+^; found 400.0975.

### Synthesis of 1

In a screw-cap vial **L** ^**2**^ (18 mg, 0.045 mmol) and copper (II) acetate (10 mg, 0.050 mmol) were combined in DMA (3.6 ml). After centrifugation, the supernatant containing **1** was collected. MeOH (8 ml) was added in one portion and the vial was left to sit for 2 days, forming crystals of **1**. ν_max_ (neat, cm^−1^): 3,631 (br, s), 1,680 (m, HC = O), 1,575 (m), 1,452 (m), 1,422 (m), 1,377 (s).

### Synthesis of 2

In a screw-cap vial **L** ^**2**^ (5 mg, 0.01 mmol) and copper (II) acetate (3 mg, 0.016 mmol) were combined in DMA (0.6 ml). After centrifugation, the supernatant containing **1** was collected. Slow vapour diffusion of diisopropylether into the solution of **1** afforded green rhombohedral crystals of **2** after 4 days. ν_max_ (neat, cm^−1^): 3,649 (br, s), 1709 (w), 1,679 m, HC = O), 1,564 (m), 1,453 (m), 1,422 (m), 1,387 (s).

### Synthesis of 3

In a screw-cap vial **L** ^**2**^ (18 mg, 0.045 mmol) and copper (II) acetate (10 mg, 0.050 mmol) were combined in DMA (4 ml). After centrifugation, the supernatant containing **1** was obtained. Terephthaldehyde (2.4 mg, 0.018 mmol, 0.4 eq) and Sc(OTf)_3_ (0.0018 mmol) were added in one portion and the mixture was left to stand for 8 h at 25 °C. After this time, the formed precipitate was, collected by centrifugation, then washed with DMSO (×5) then MeOH (×4) before being dried under vacuum to afford **3** as a solid. ν_max_ (neat, cm^−1^): 1,678 (m, HC=O), 1,579 (m), 1,546 (s, C=N, 1,451 (m), 1,425 (m), 1,388 (s).

## Data Availability

The X-ray crystallographic datasets presented in this study can be found in online repositories. The names of the repository/repositories and accession number(s) can be found below: https://www.ccdc.cam.ac.uk/solutions/csd-system/components/csd/, 2072629, 2072630.
