# Supplementary material for: Assembly and Covalent Cross-Linking of an Amine-Functionalised Metal-Organic Cage
Source: Front Chem. 2021 May 25;9:696081. doi: 10.3389/fchem.2021.696081 (PMC8185198; doi:10.3389/fchem.2021.696081)
Supplement: Supplementary file 1 [file DataSheet1.docx]

Supporting information

Assembly and covalent cross-linking of an amine-functionalised metal-organic cage

Matthew L. Schneider,^a^ Adrian Markwell-Heys^a^, Oliver M. Linder-Patton^a^ and Witold M. Bloch*^a^

^a^Department of Chemistry, The University of Adelaide, Adelaide, Australia.

**Contents**

[1 NMR spectroscopy 2](#_Toc71031326)

[1.1 ^1^H NMR spectrum of L^2^ 2](#_Toc71031327)

[1.2 Dissolution of MOF 2 via sonication 2](#_Toc71031328)

[1.3 ^1^H – ^1^H COSY of a digested sample of 3 3](#_Toc71031329)

[2 UV- Vis spectroscopy 3](#_Toc71031330)

[3 IR spectroscopy 4](#_Toc71031331)

[4 Powder X-ray diffraction (PXRD) 6](#_Toc71031332)

[5 Thermal gravimetric analysis (TGA) 8](#_Toc71031333)

[6 Gas adsorption 10](#_Toc71031334)

[7 SEM and EDX data of 1, 2 and 3. 13](#_Toc71031335)

[8 X-ray crystallography 14](#_Toc71031336)

[8.1 General methods 14](#_Toc71031337)

[8.2 Specific refinement details for 2 14](#_Toc71031338)

[8.3 Thermal ellipsoid plots 16](#_Toc71031339)

[8.4 Checkcif reports 17](#_Toc71031340)

[9 References 22](#_Toc71031341)

# NMR spectroscopy

## ^1^H NMR spectrum of L^2^


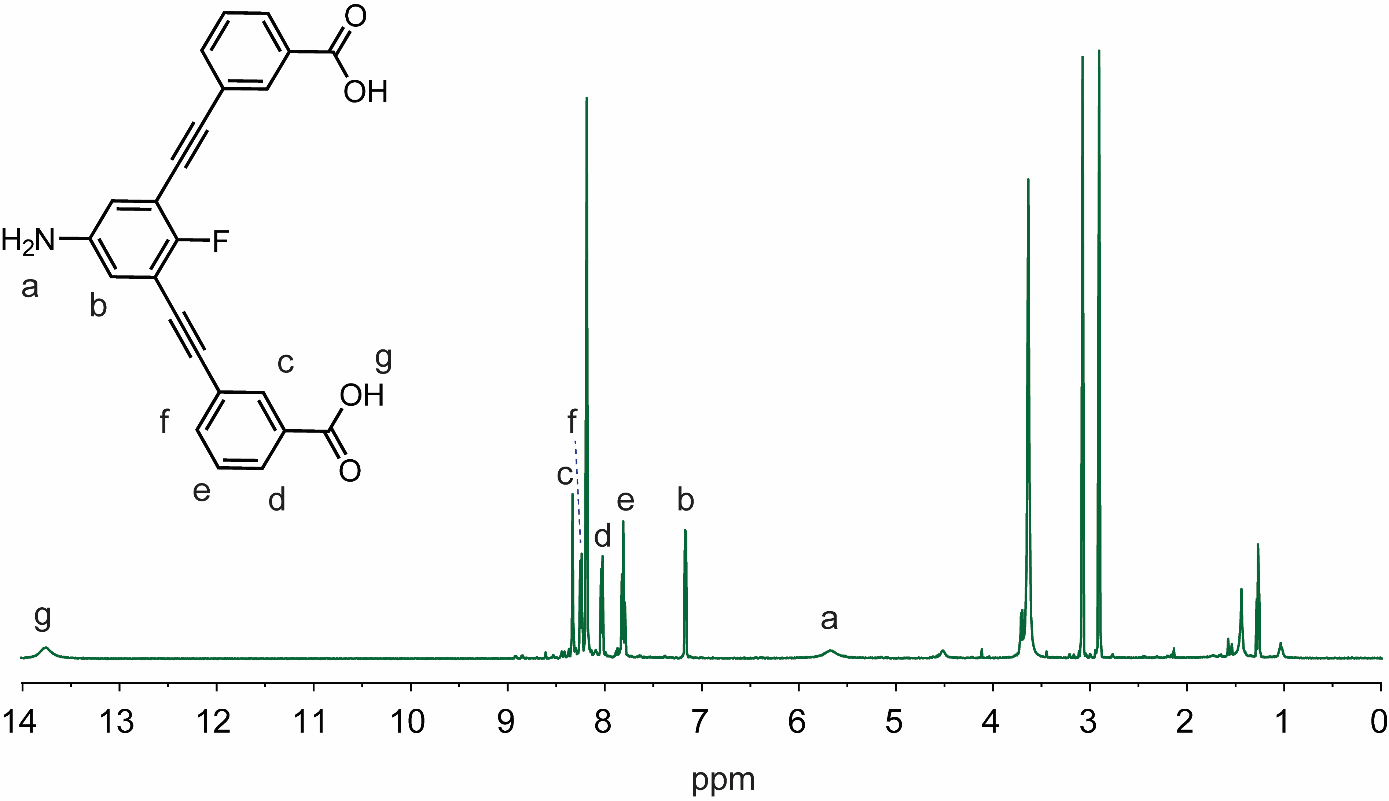


1. ^1^H NMR spectrum (DMF-d_7_ / 500 MHz) of **L^2^**.

## Dissolution of MOF 2 via sonication


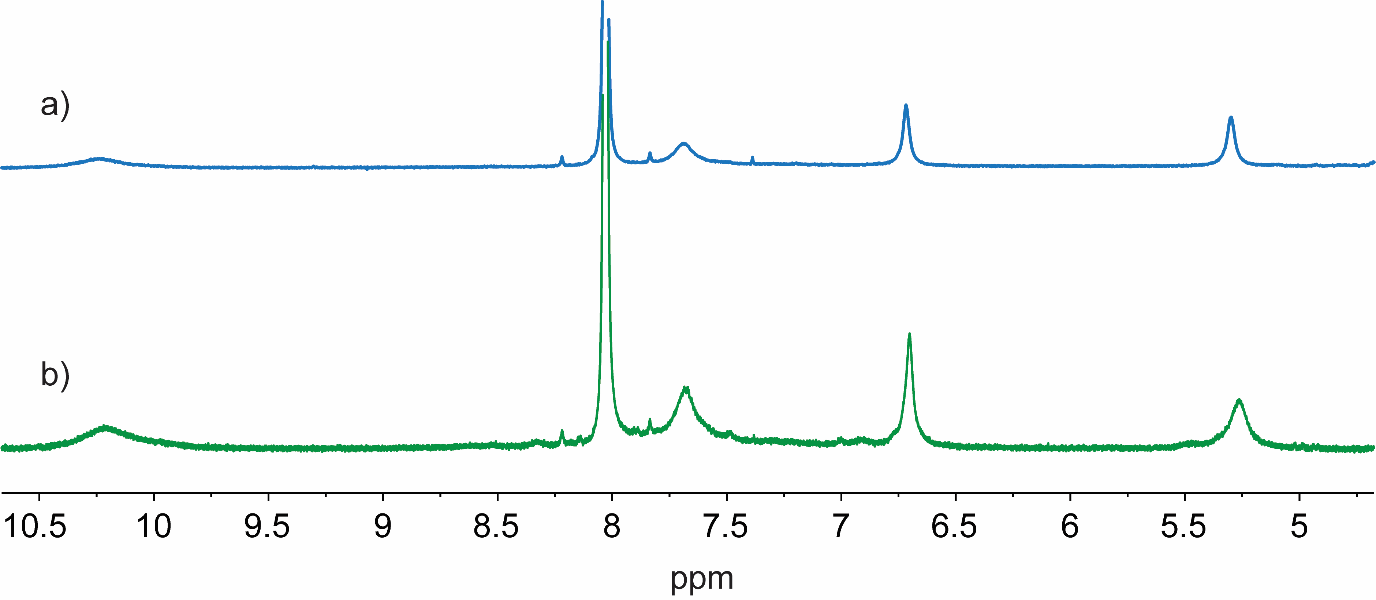


1. ^1^H NMR spectra (500 MHz, DMF-d_7_) of a) sonicated sample of **2** in DMF-d_7_ and b) discrete cage **1** formed directly in DMF-d_7_. The dissolution of MOF **2** upon sonication in DMF-d_7_ reveals ^1^H NMR resonances directly correlating to the discrete cage **1**.

## ^1^H – ^1^H COSY of a digested sample of 3


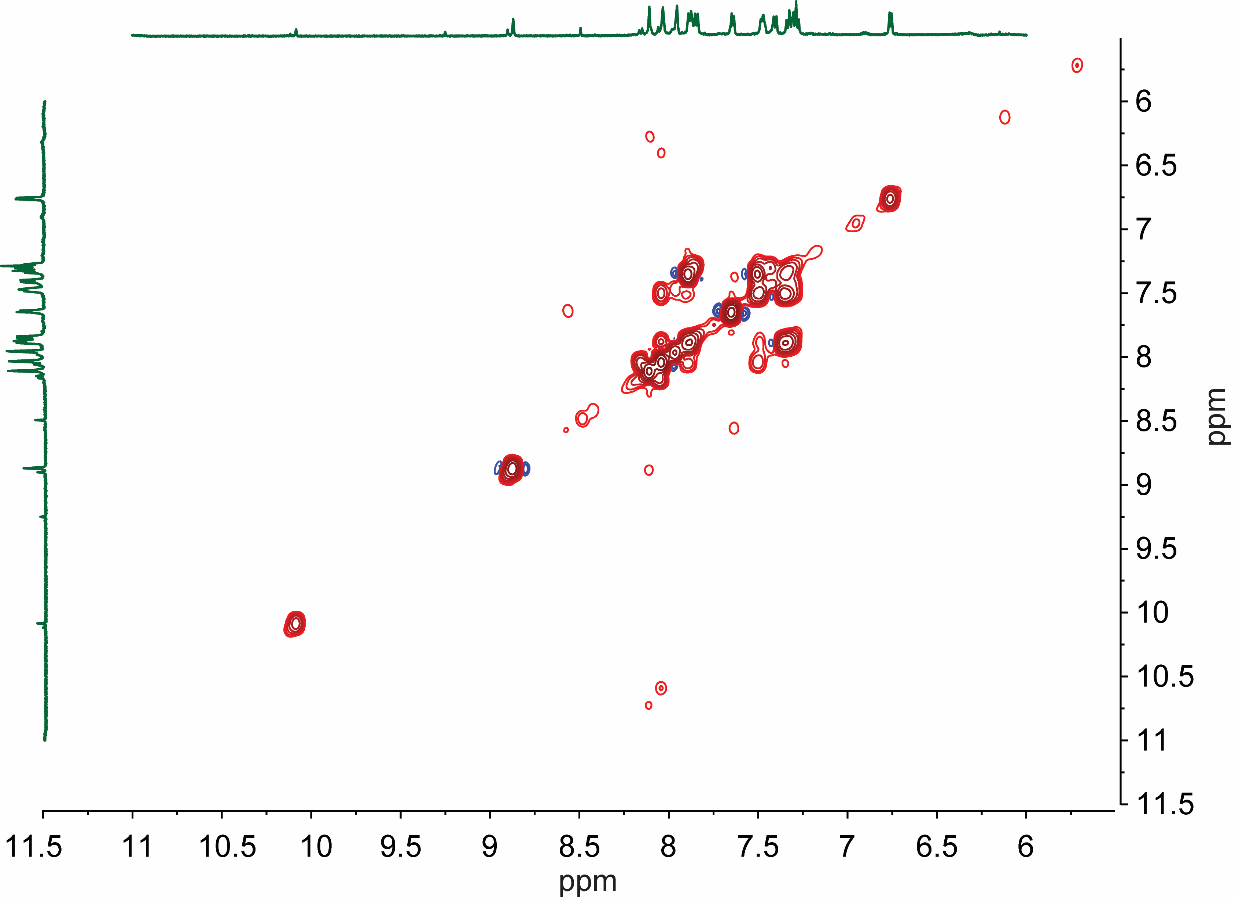


1. ^1^H-^1^H COSY NMR spectrum (500 MHz, DMSO-d_6_) of an EGTA digestion of cross-linked MOC polymer **3**.

# UV- Vis spectroscopy





1. UV-Vis spectrum of **L^2^**, **1** and **Cu(OAc)_2_** in DMF indicating formation of the cage species **1** in solution. Shifting of the maxima absorption peak in the copper paddlewheel region (600 – 800) is consistent with the formation of the desired Cu_4_L_4_ copper paddlewheel cage.





1. UV-Vis spectrum of **L^2^**, **1** and **Cu(OAc)_2_** in DMF indicating formation of the cage species **1** in solution. Shifting of the maxima absorption peak in the copper paddlewheel region (600 – 800) is consistent with the formation of the desired Cu_4_L_4_ copper paddlewheel cage.

# IR spectroscopy





1. IR spectrum of MOC **1**.





1. IR spectrum of cross-linked polymer **3**. The pronounced imine stretch at 1622 cm^-1^ corresponds to the C=N stretching mode associated with the cross-linked material.





1. IR spectrum of MOF **2**.

# Powder X-ray diffraction (PXRD)


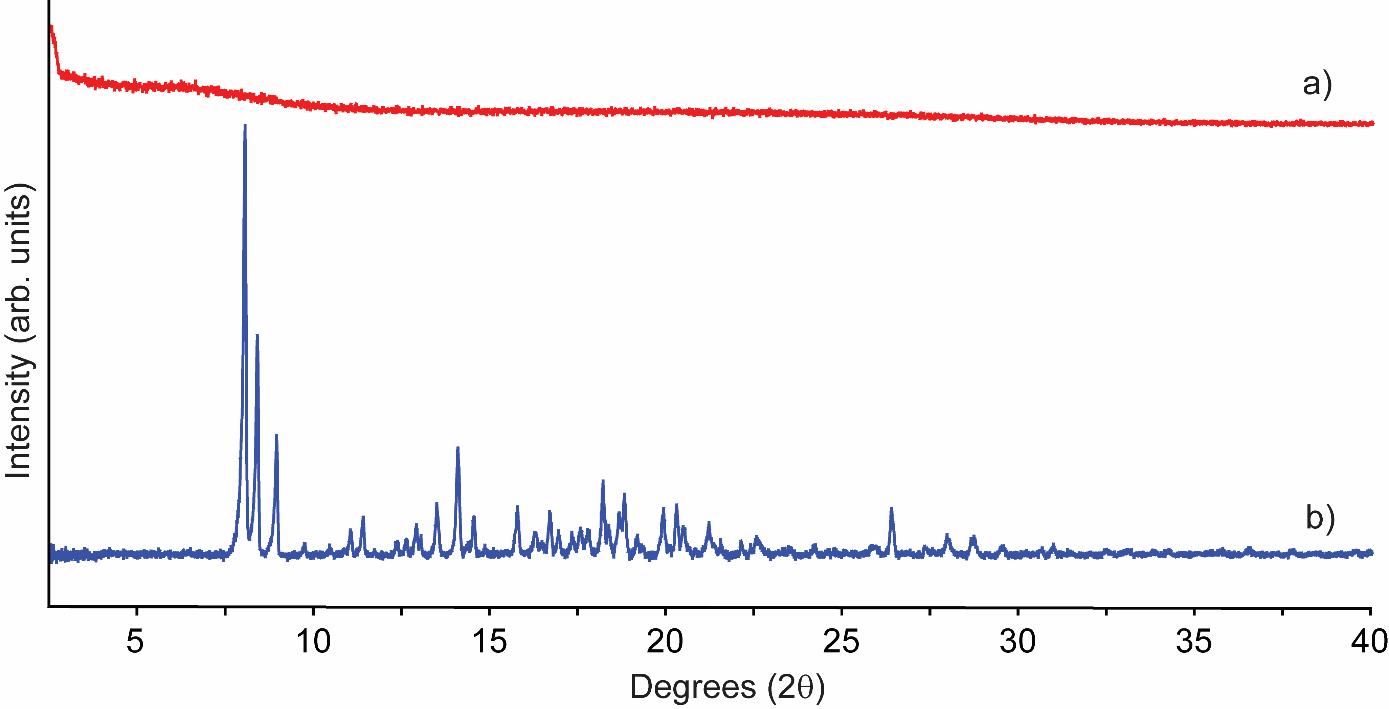


1. Powder X-ray diffraction (PXRD) patterns of a) an activated sample of **2** and b) an as-synthesised form of **2**.


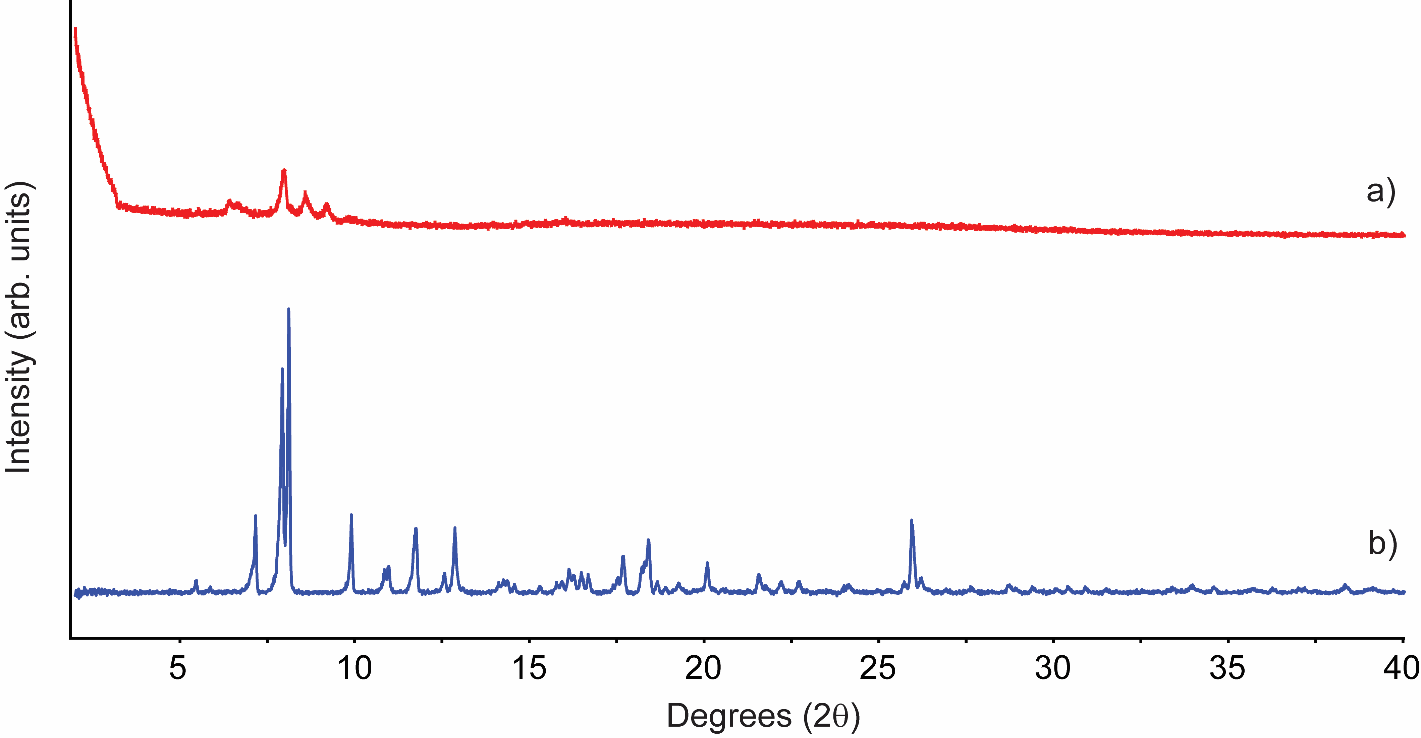


1. Powder x-ray diffraction (PXRD) patterns of a) an activated sample of **1** and b) an as-synthesised sample of **1**.


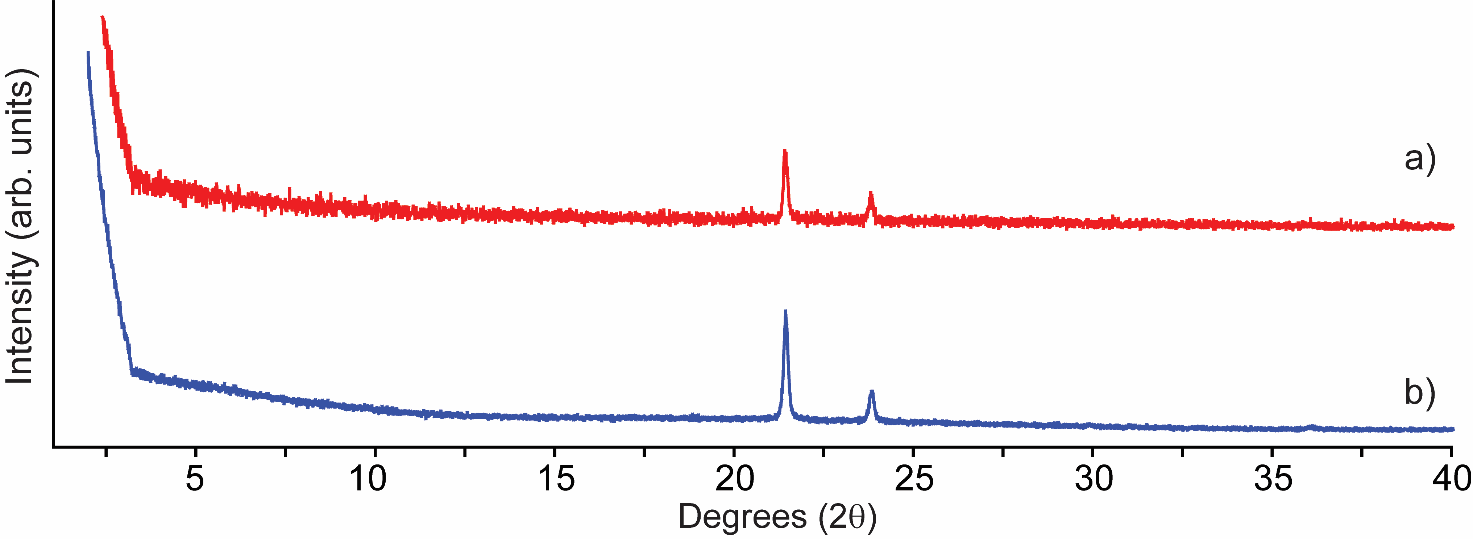


1. Powder x-ray diffraction (PXRD) of a) Activated sample of **3** and b) an as-synthesised sample of **3**.


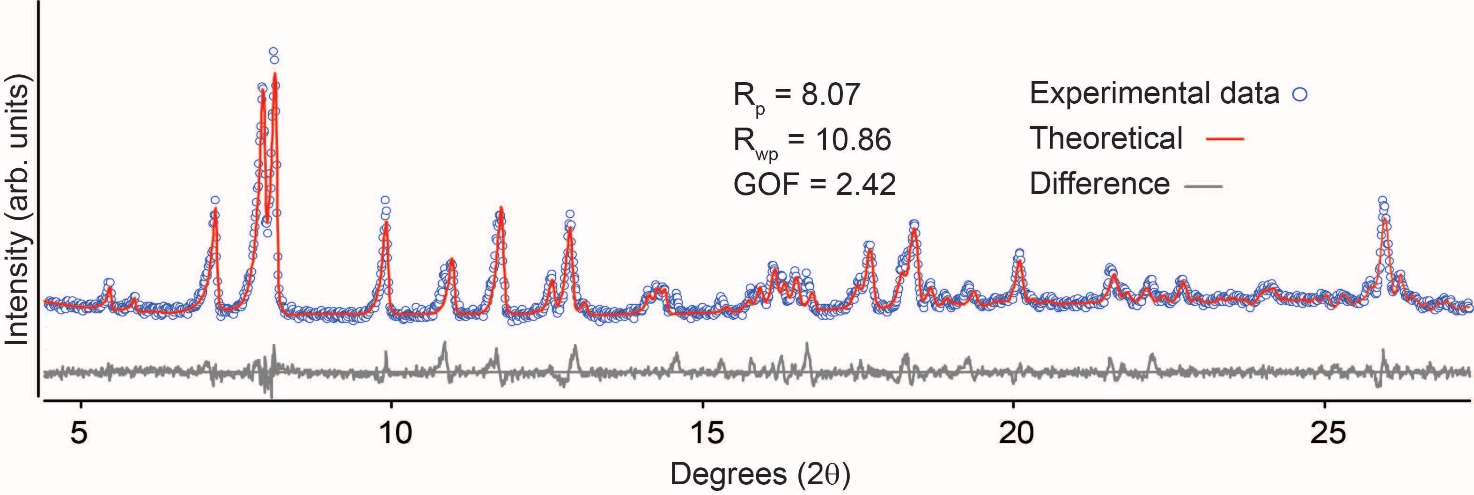


1. Rietveld PXRD refinement of a bulk sample of **1**.


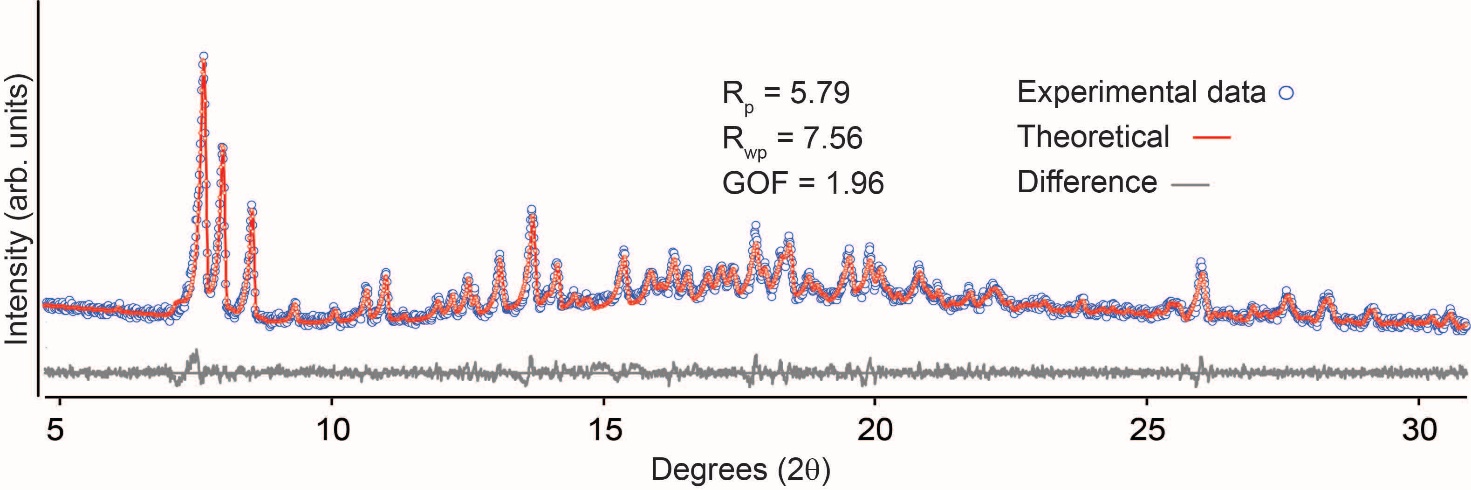


1. Rietveld PXRD refinement of a bulk sample of **2**.


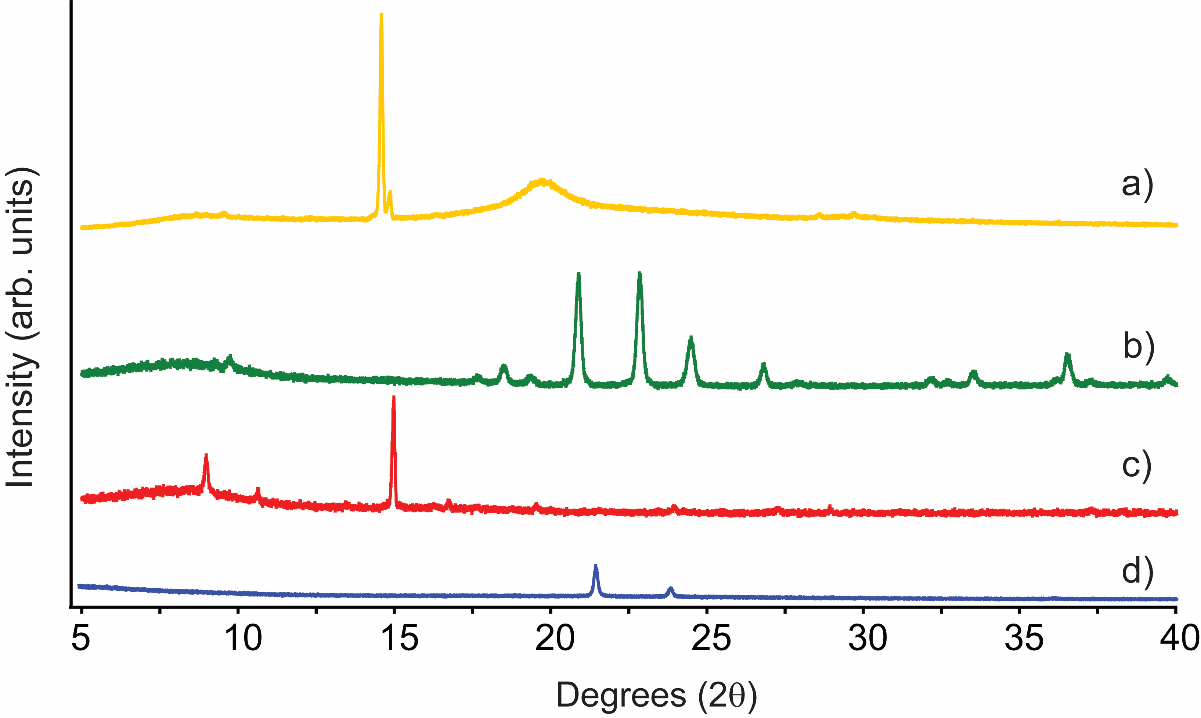


1. Powder X-ray diffraction (PXRD) patterns of a) Cu(OAc)_2_, b) Sc(OTf)_3_, c) ground sample of Sc(OTf)_3_ + Cu(OAc)_2_ mixed together and d) activated sample of **3**. This indicates that the diffraction peaks at 21° and 23° observed in **3** do not originate from either the Cu^II^ source for cage formation, or the Sc(OTf)_3_ utilised for catalysing the cross-linking reaction.

# Thermal gravimetric analysis (TGA)





1. TGA-DSC trace of the solid sample of **2** (black line = % weight loss, blue line = DSC trace). Onset of decomposition occurs at ~ 280 °C.





1. TGA-DSC trace of the solid sample of **3** (black line = % weight loss, blue line = DSC trace). Onset of decomposition occurs at ~ 280 °C.





1. TGA-DSC trace of the solid sample of **1** (black line = % weight loss, blue line = DSC trace). Onset of decomposition occurs at ~ 280 °C.

# Gas adsorption

**1** and **3** were washed with MeOH ( x 6) over 18 hours. **2** was washed with acetone ( x 6 ) over 18 hours. All samples activated under high vacuum for 6 hours at 90°C, then 8 hours at 60° prior to the experiment commencement.





1. 77k N_2_ adsorption isotherm of **2** & **3**.





1. 77k N_2_ adsorption isotherm of **1**.





1. 195k CO_2_ isotherm of **2** and **3**.





1. 195k CO_2_ isotherm of **1**.


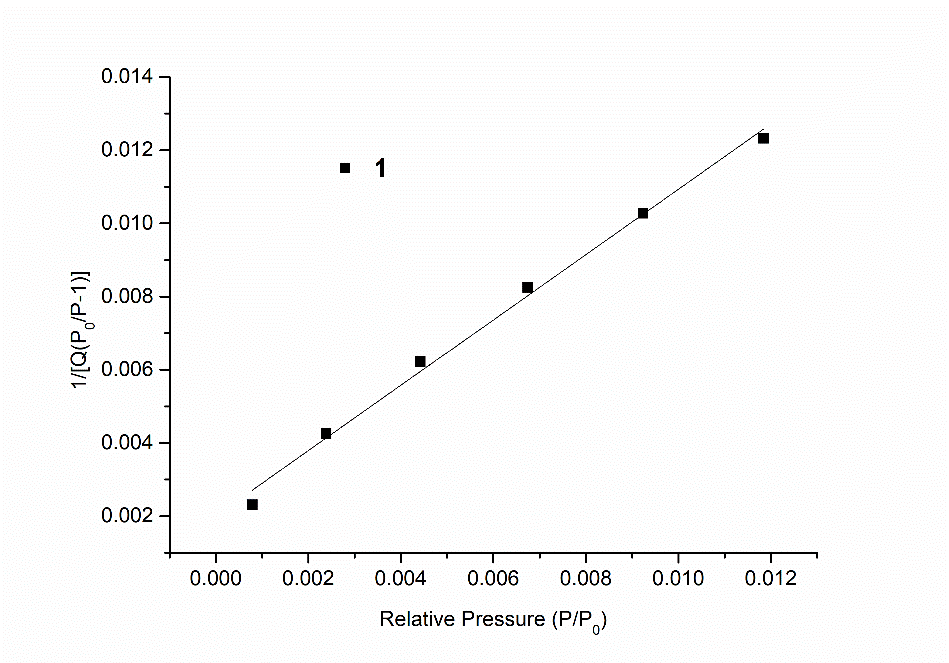

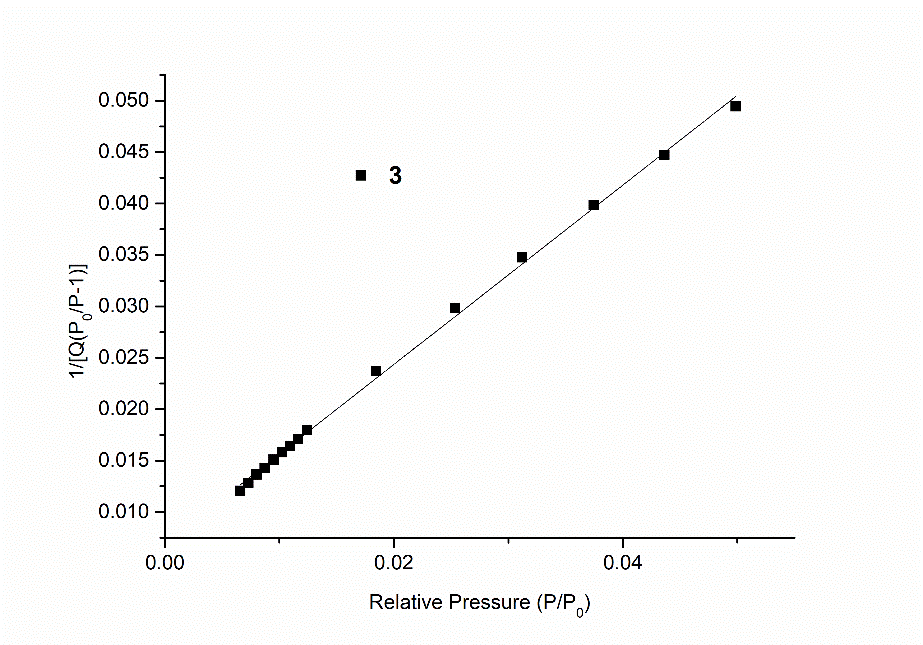

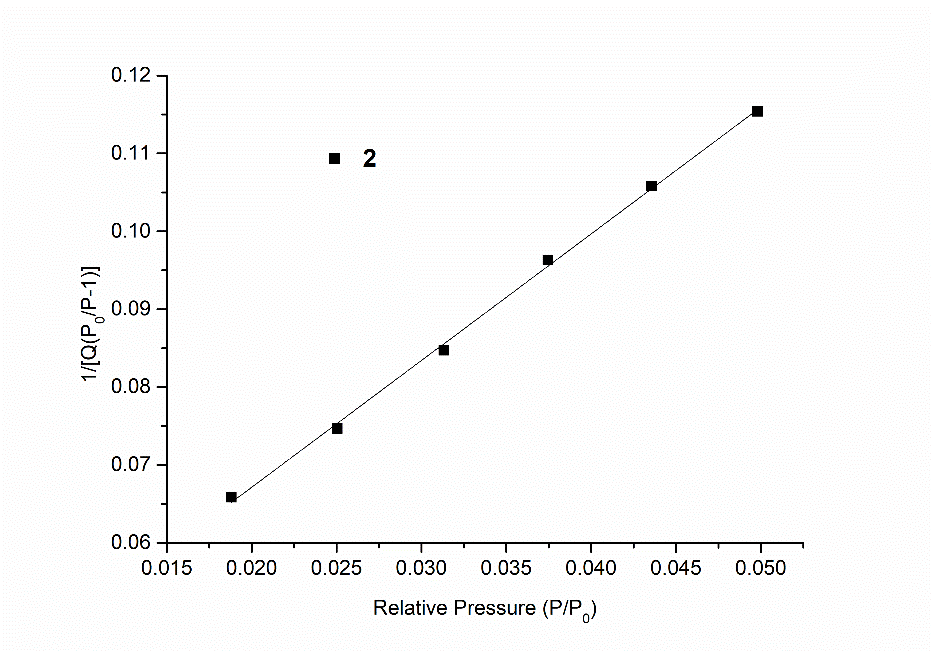


1. Derivation of the BET surface area from the 195 K CO_2_ adsorption isotherms for **1**, **2** and **3**.

**Table S1:** BET surface areas derived from the 195k CO_2_ isotherm of **1**, **2** and **3.**

| **Sample** | **BET (m^2^ / g)** |
| --- | --- |
| **1** | **95 ± 3** |
| **2** | **51 ± 1** |
| **3** | **97 ± 1** |

# SEM and EDX data of 1, 2 and 3.

Scanning Electron Microscope (SEM) images were collected on a Phillips XL30/Quanta 450 scanning electron microscope in secondary electron mode, (spot size 3 and 10 KeV). Electron Dispersive X-ray Analysis was collected with an Oxford Instruments Ultim Max 170 EDX attachment on the Phillips XL30/Quanta 450 (spot size 4, 15 KeV). Samples for SEM analysis were dry loaded onto adhesive carbon tabs on aluminium stubs and carbon coated (5 nm) prior to analysis.


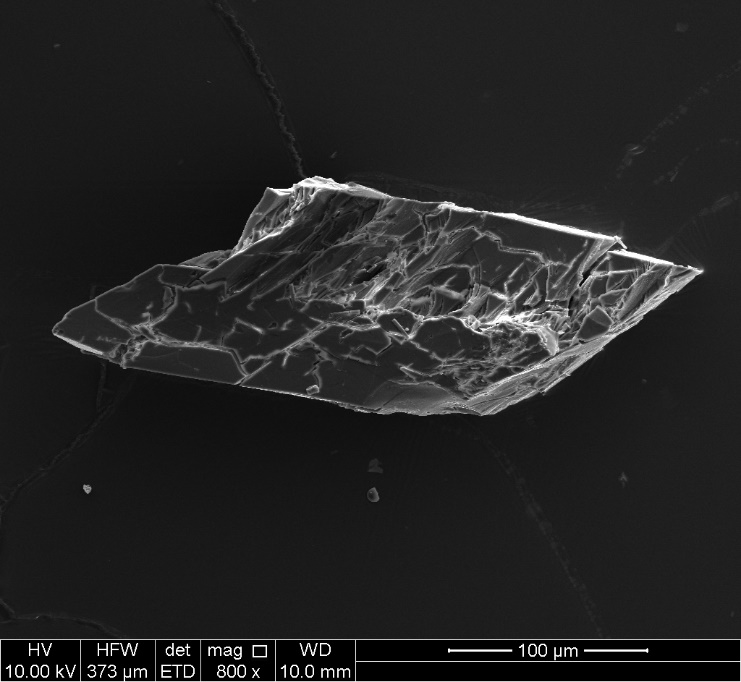

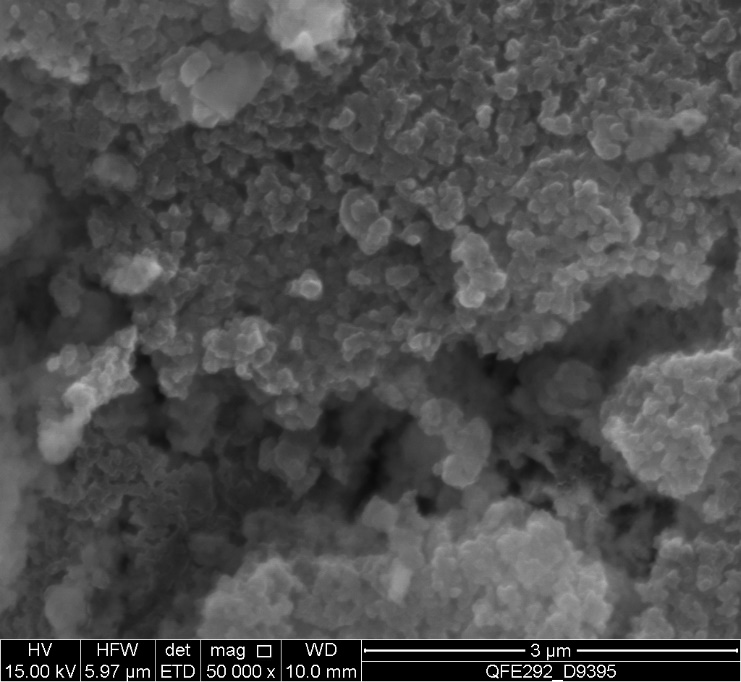


a)


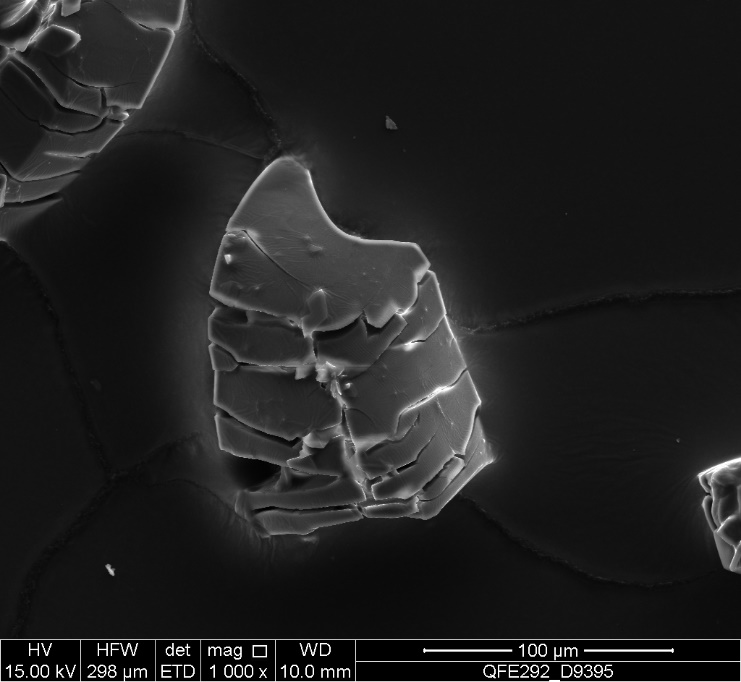


b)

c)

1. SEM images of a) **3**, b) **2** and c) **1**.


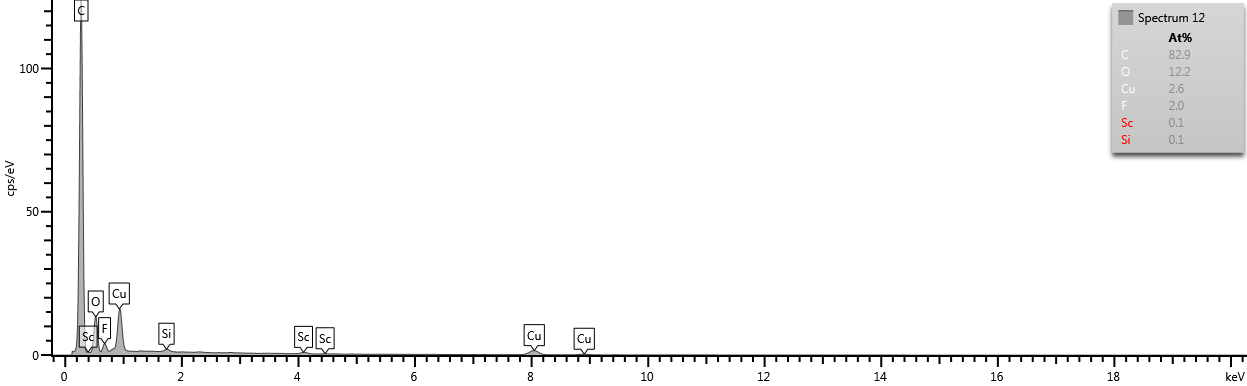

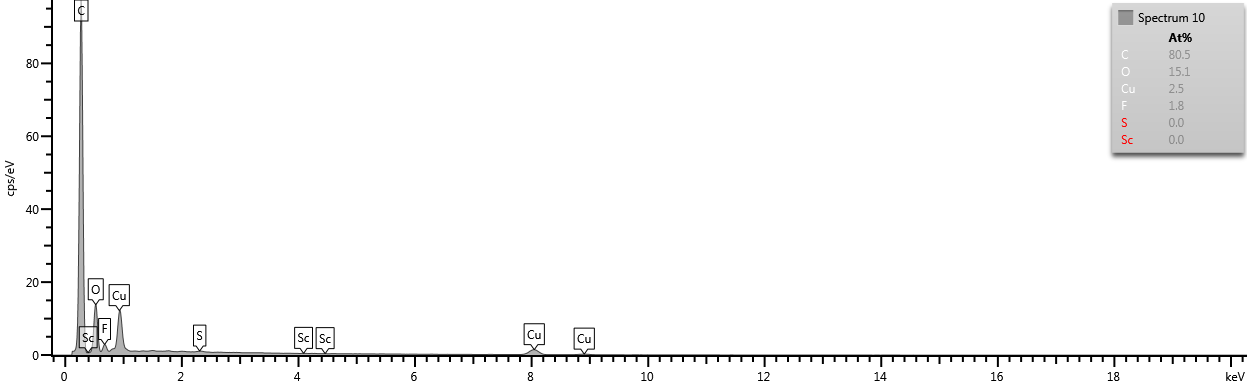


a)

b)


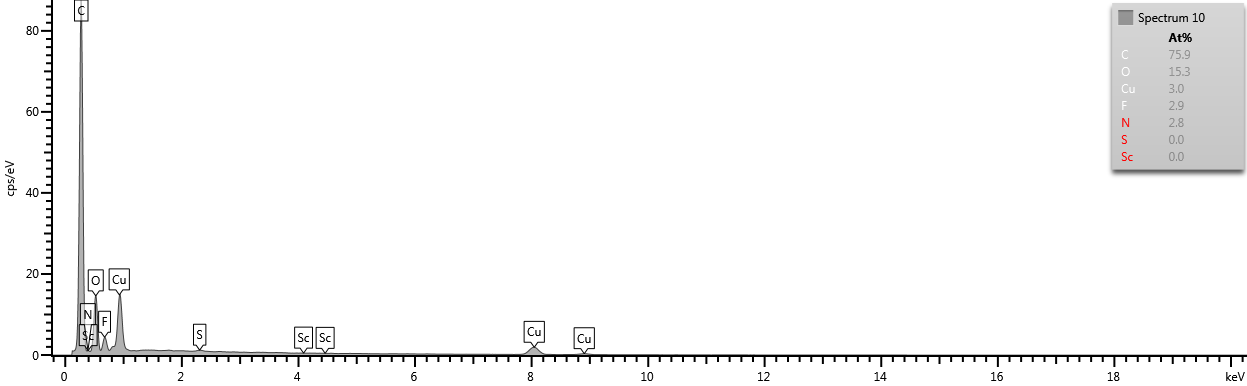


c)

1. EDX graphs confirming the presence of copper and absence of scandium in samples a) **2**, b) **3** and c) **1**.

# X-ray crystallography

## General methods

Single crystals were mounted in paratone-N oil on a plastic loop. X-ray diffraction data for **1** and **2** was collected at 100(2) K on the MX-1 or MX-2 beamline of the Australian Synchrotron.(Cowieson et al. 2015)^,^(Aragao et al. 2018) Data sets were corrected for absorption using a multi-scan method, and structures were solved by direct methods using SHELXT(MAYRA 2013) and refined with SHELXL(García Reyes 2013) and ShelXle(Hubschle, Sheldrick, and Dittrich 2011) as a graphical user interface. All non-hydrogen atoms were refined anisotropically and hydrogen atoms were included as invariants at geometrically estimated positions. The contribution of the electron density from disordered, pore-bound solvent molecules, which could not be modelled with discrete atomic positions were handled using the SQUEEZE(A. L. Spek 2015) routine in PLATON,(A. Spek 2009) which strongly improved all figures of merit (FOM). X-ray experimental data is given in Table S2.

## Specific refinement details for 2

Stereochemical restraints for DMA solvent molecules (residue defined as DMA) were generated by the GRADE program using the GRADE Web Server (http://grade.globalphasing.org) and applied in the refinement. This helped to resolve the disorder of the coordinated DMA molecules, particularly the DMA ligand bound to the interior of the Cu_2_ paddlewheel. The refinement of ADP's for carbon, nitrogen and oxygen atoms was supported by similarity restraints (SIMU).(Thorn, Dittrich, and Sheldrick 2012)

**Table S2:** X-ray experimental data **1** and **2**

| Compound | **1** | **2** |
| --- | --- | --- |
| CCDC number | 2072629 | 2072630 |
| Empirical formula | C_97_H_51_Cu_4_F_4_N_4_O_20_ | C_60_H_51_Cu_2_F_2_N_5_O_11_ |
| Formula weight | 1922.57 | 1183.14 |
| Crystal system | Triclinic | Monoclinic |
| Space group | *P*-1 | *P*2**_1_**/c |
| *a* (Å) | 13.999(3) | 15.789(3) |
| *b* (Å) | 15.580(3) | 16.391(3) |
| *c* (Å) | 16.461(3) | 29.504(6) |
| α (º) | 83.64(3)° | 90 |
| β (º) | 73.19(3)° | 95.95(3) |
| γ (º) | 68.86(3)° | 90 |
| Volume (Å^3^) | 3205.4(14) | 7594(3) |
| *Z* | 1 | 4 |
| Density (calc.) (Mg/m^3^) | 0.996 | 1.035 |
| Absorption coefficient (mm^-1^) | 0.711 | 0.613 |
| F(000) | 973 | 2440 |
| Crystal size (mm^3^) | 0.22x0.18 x0.09 | 0.18x0.13x0.08 |
| θ range for data collection (º) | 1.292 to 25.681 | 1.297 to 27.111 |
| Reflections collected | 38886 | 96579 |
| Observed reflections [R(int)] | 11062 [0.0496] | 15737 [0.0179] |
| Goodness-of-fit on F^2^ | 1.067 | 1.051 |
| R_1_ [I>2σ(Ι)] | 0.0524 | 0.0725 |
| wR_2_ (all data) | 0.1723 | 0.2423 |
| Largest diff. peak and hole (e.Å-3) | 0.845 and -0.582 | 1.165 and -0.928 |
| Data / restraints / parameters | 11062 / 0 / 587 | 15737 / 752 / 841 |

## Thermal ellipsoid plots


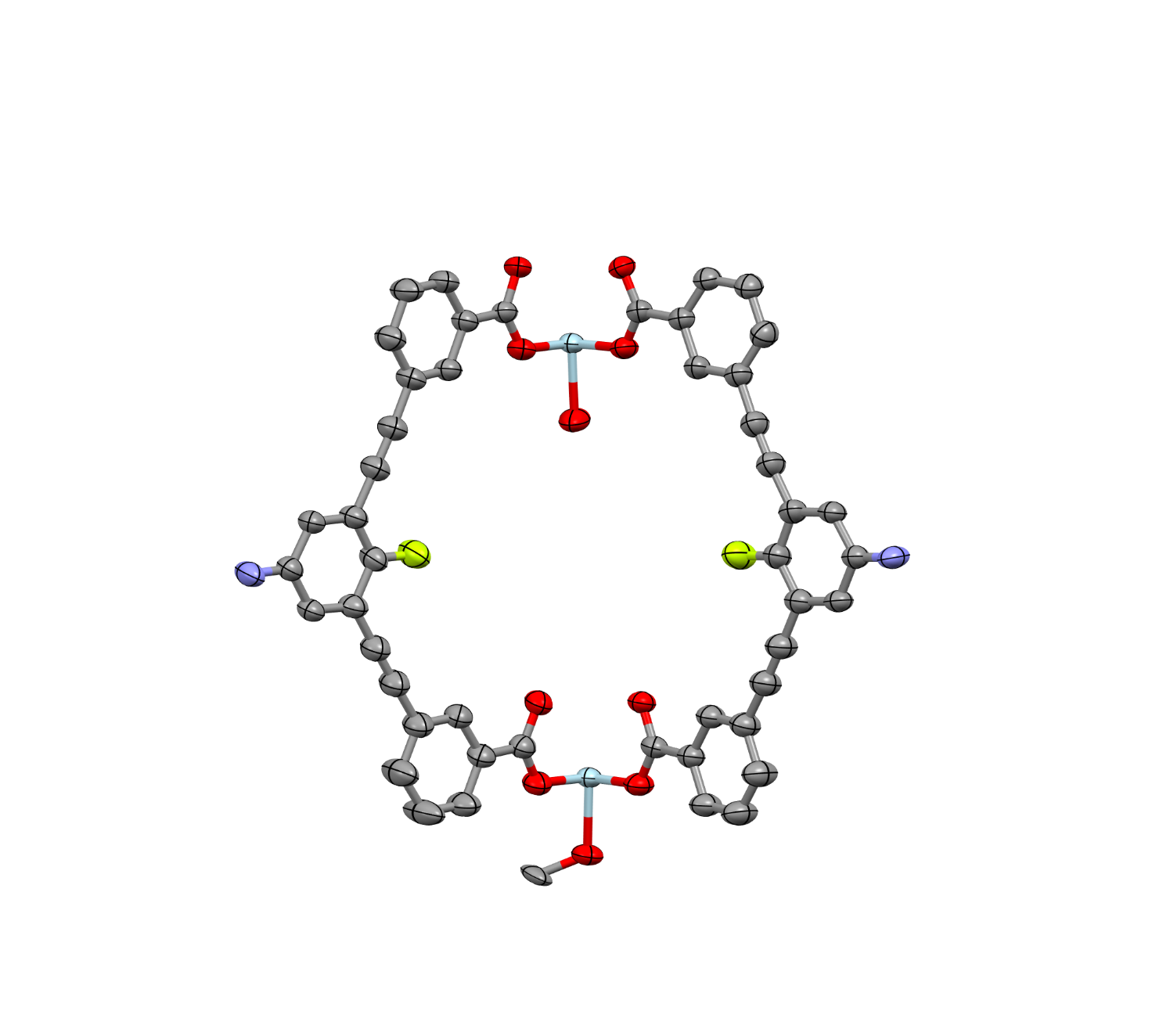


1. The asymmetric unit of the X-ray structures of **1** with all non-hydrogen atoms shown as ellipsoids at the 50% probability level (hydrogen atoms omitted for clarity). C = grey, N = purple, O = red, Cu = light blue.


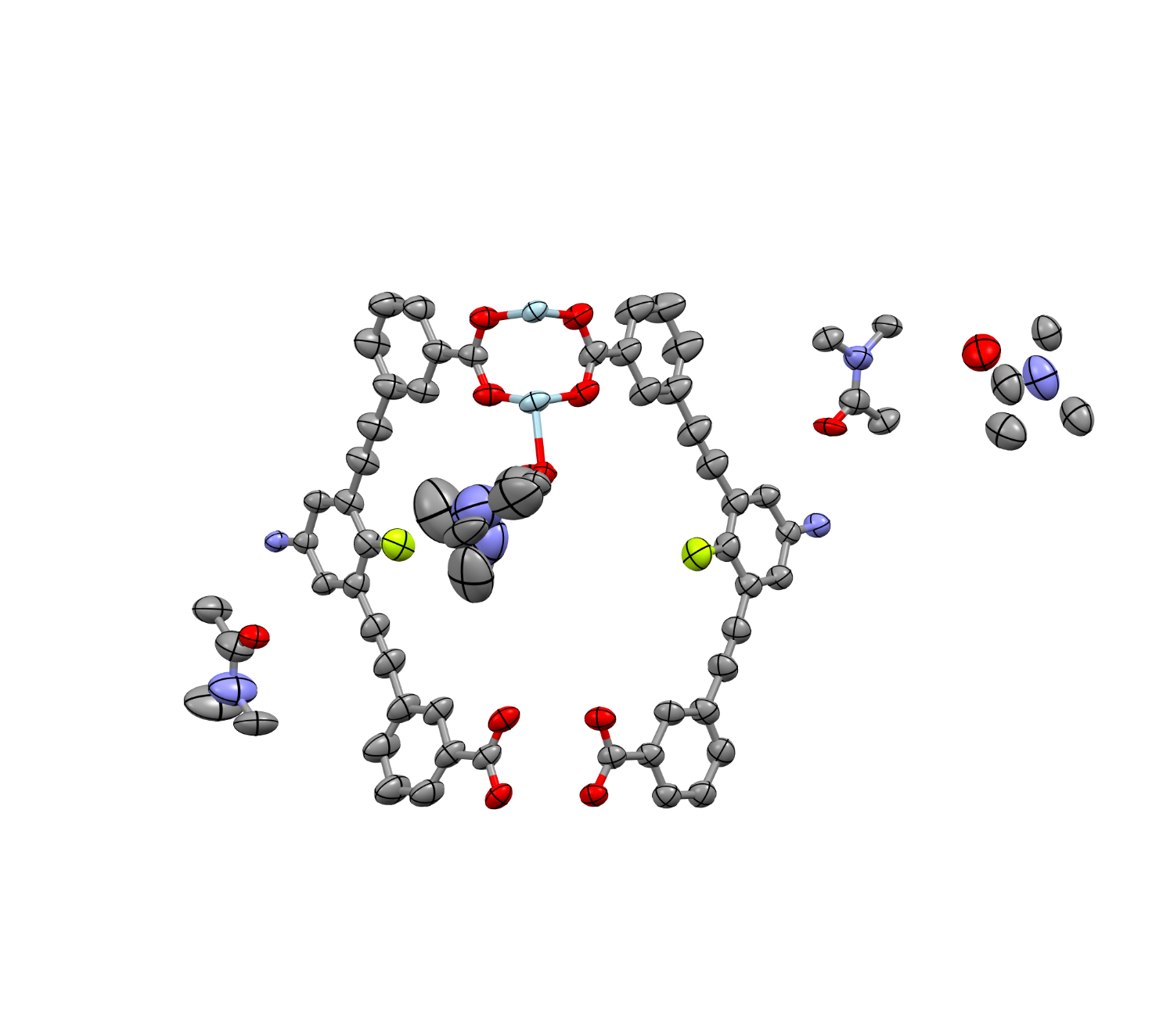


1. The asymmetric unit of the X-ray structures of **2** with all non-hydrogen atoms shown as ellipsoids at the 50% probability level (hydrogen atoms omitted for clarity). C = grey, N = purple, O = red, Cu = light blue.

## Checkcif reports

**Datablock: 1**

| Bond precision: | C-C = 0.0047 A | Wavelength=0.71073 |
| --- | --- | --- |

| Cell: | a=13.999(3) | b=15.580(3) | c=16.461(3) |
| --- | --- | --- | --- |
|  | alpha=83.64(3) | beta=73.19(3) | gamma=68.86(3) |
| Temperature: | 100 K |  |  |

|  | Calculated | Reported |
| --- | --- | --- |
| Volume | 3205.4(14) | 3205.4(14) |
| Space group | P -1 | P -1 |
| Hall group | -P 1 | -P 1 |
| Moiety formula | C97 H51 Cu4 F4 N4 O20 [+ solvent] | ? |
| Sum formula | C97 H51 Cu4 F4 N4 O20 [+ solvent] | C97 H51 Cu4 F4 N4 O20 |
| Mr | 1922.62 | 1922.57 |
| Dx,g cm-3 | 0.996 | 0.996 |
| Z | 1 | 1 |
| Mu (mm-1) | 0.711 | 0.711 |
| F000 | 973.0 | 973.0 |
| F000' | 974.75 |  |
| h,k,lmax | 17,18,20 | 17,18,20 |
| Nref | 12162 | 11062 |
| Tmin,Tmax | 0.858,0.938 |  |
| Tmin' | 0.855 |  |

| Correction method= Not given |  |
| --- | --- |

| Data completeness= 0.910 | Theta(max)= 25.681 |
| --- | --- |

| R(reflections)= 0.0524( 9660) | wR2(reflections)= 0.1723( 11062) |
| --- | --- |

| S = 1.067 | Npar= 587 |
| --- | --- |

The following ALERTS were generated. Each ALERT has the format

**test-name_ALERT_alert-type_alert-level**.

Click on the hyperlinks for more details of the test.


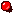
**Alert level A**

[PLAT029_ALERT_3_A](javascript:makeHelpWindow(%22PLAT029.html%22)) _diffrn_measured_fraction_theta_full value Low . 0.911 Why?

|  | **Author Response: due to geometry constraints of the endstation only a single omega sweep was able to be collected at the Australian Synchrotron MX1 beamline. The triclinic crystals were sensitive to solvent loss and a more complete data set could not be collected.** |
| --- | --- |


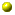
**Alert level C**

[PLAT052_ALERT_1_C](javascript:makeHelpWindow(%22PLAT052.html%22)) Info on Absorption Correction Method Not Given Please Do !

[PLAT241_ALERT_2_C](javascript:makeHelpWindow(%22PLAT241.html%22)) High 'MainMol' Ueq as Compared to Neighbors of C20_1 Check

[PLAT242_ALERT_2_C](javascript:makeHelpWindow(%22PLAT242.html%22)) Low 'MainMol' Ueq as Compared to Neighbors of Cu01 Check

[PLAT250_ALERT_2_C](javascript:makeHelpWindow(%22PLAT250.html%22)) Large U3/U1 Ratio for Average U(i,j) Tensor .... 2.1 Note

[PLAT420_ALERT_2_C](javascript:makeHelpWindow(%22PLAT420.html%22)) D-H Bond Without Acceptor N1_1 --H1B_1 . Please Check

[PLAT420_ALERT_2_C](javascript:makeHelpWindow(%22PLAT420.html%22)) D-H Bond Without Acceptor N1_2 --H1B_2 . Please Check


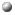
**Alert level G**

[ABSMU01_ALERT_1_G](javascript:makeHelpWindow(%22ABSMU_01.html%22)) Calculation of _exptl_absorpt_correction_mu

not performed for this radiation type.

[PLAT007_ALERT_5_G](javascript:makeHelpWindow(%22PLAT007.html%22)) Number of Unrefined Donor-H Atoms .............. 4 Report

[PLAT154_ALERT_1_G](javascript:makeHelpWindow(%22PLAT154.html%22)) The s.u.'s on the Cell Angles are Equal ..(Note) 0.03 Degree

[PLAT300_ALERT_4_G](javascript:makeHelpWindow(%22PLAT300.html%22)) Atom Site Occupancy of C3 Constrained at 0.5 Check

**And 3 other PLAT300 Alerts**

[PLAT300_ALERT_4_G](javascript:makeHelpWindow(%22PLAT300.html%22)) Atom Site Occupancy of H3A Constrained at 0.5 Check

[PLAT300_ALERT_4_G](javascript:makeHelpWindow(%22PLAT300.html%22)) Atom Site Occupancy of H3B Constrained at 0.5 Check

[PLAT300_ALERT_4_G](javascript:makeHelpWindow(%22PLAT300.html%22)) Atom Site Occupancy of H3C Constrained at 0.5 Check

[PLAT301_ALERT_3_G](javascript:makeHelpWindow(%22PLAT301.html%22)) Main Residue Disorder ..............(Resd 1 ) 1% Note

[PLAT371_ALERT_2_G](javascript:makeHelpWindow(%22PLAT371.html%22)) Long C(sp2)-C(sp1) Bond C6_1 - C8_1 . 1.44 Ang.

**And 7 other PLAT371 Alerts**

[PLAT371_ALERT_2_G](javascript:makeHelpWindow(%22PLAT371.html%22)) Long C(sp2)-C(sp1) Bond C9_1 - C10_1 . 1.44 Ang.

[PLAT371_ALERT_2_G](javascript:makeHelpWindow(%22PLAT371.html%22)) Long C(sp2)-C(sp1) Bond C12_1 - C16_1 . 1.42 Ang.

[PLAT371_ALERT_2_G](javascript:makeHelpWindow(%22PLAT371.html%22)) Long C(sp2)-C(sp1) Bond C17_1 - C18_1 . 1.44 Ang.

[PLAT371_ALERT_2_G](javascript:makeHelpWindow(%22PLAT371.html%22)) Long C(sp2)-C(sp1) Bond C6_2 - C8_2 . 1.43 Ang.

[PLAT371_ALERT_2_G](javascript:makeHelpWindow(%22PLAT371.html%22)) Long C(sp2)-C(sp1) Bond C9_2 - C10_2 . 1.44 Ang.

[PLAT371_ALERT_2_G](javascript:makeHelpWindow(%22PLAT371.html%22)) Long C(sp2)-C(sp1) Bond C12_2 - C16_2 . 1.43 Ang.

[PLAT371_ALERT_2_G](javascript:makeHelpWindow(%22PLAT371.html%22)) Long C(sp2)-C(sp1) Bond C17_2 - C18_2 . 1.43 Ang.

[PLAT606_ALERT_4_G](javascript:makeHelpWindow(%22PLAT606.html%22)) Solvent Accessible VOID(S) in Structure ........ ! Info

[PLAT720_ALERT_4_G](javascript:makeHelpWindow(%22PLAT720.html%22)) Number of Unusual/Non-Standard Labels .......... 86 Note

[PLAT794_ALERT_5_G](javascript:makeHelpWindow(%22PLAT794.html%22)) Tentative Bond Valency for Cu01 (II) . 2.14 Info

[PLAT802_ALERT_4_G](javascript:makeHelpWindow(%22PLAT802.html%22)) CIF Input Record(s) with more than 80 Characters 1 Info

[PLAT869_ALERT_4_G](javascript:makeHelpWindow(%22PLAT869.html%22)) ALERTS Related to the Use of SQUEEZE Suppressed ! Info

[PLAT883_ALERT_1_G](javascript:makeHelpWindow(%22PLAT883.html%22)) No Info/Value for _atom_sites_solution_primary . Please Do !

[PLAT941_ALERT_3_G](javascript:makeHelpWindow(%22PLAT941.html%22)) Average HKL Measurement Multiplicity ........... 3.5 Low

[PLAT965_ALERT_2_G](javascript:makeHelpWindow(%22PLAT965.html%22)) The SHELXL WEIGHT Optimisation has not Converged Please Check

**Datablock: 2**

| Bond precision: | C-C = 0.0057 A | Wavelength=0.71073 |
| --- | --- | --- |

| Cell: | a=15.789(3) | b=16.391(3) | c=29.504(6) |
| --- | --- | --- | --- |
|  | alpha=90 | beta=95.95(3) | gamma=90 |
| Temperature: | 100 K |  |  |

|  | Calculated | Reported |
| --- | --- | --- |
| Volume | 7594(3) | 7594(3) |
| Space group | P 21/c | P 21/c |
| Hall group | -P 2ybc | -P 2ybc |
| Moiety formula | C52 H33 Cu2 F2 N3 O9, 2(C2 H4.50 N0.50 O0.50), C2 H4.50 N0.50 O | ? |
| Sum formula | C60 H51 Cu2 F2 N5 O11 [+ solvent] | C60 H51 Cu2 F2 N5 O11 |
| Mr | 1183.16 | 1183.14 |
| Dx,g cm-3 | 1.035 | 1.035 |
| Z | 4 | 4 |
| Mu (mm-1) | 0.613 | 0.613 |
| F000 | 2440.0 | 2440.0 |
| F000' | 2443.65 |  |
| h,k,lmax | 20,21,37 | 20,21,37 |
| Nref | 16768 | 15737 |
| Tmin,Tmax | 0.909,0.952 |  |
| Tmin' | 0.896 |  |

| Correction method= Not given |  |
| --- | --- |

| Data completeness= 0.939 | Theta(max)= 27.111 |
| --- | --- |

| R(reflections)= 0.0725( 14057) | wR2(reflections)= 0.2423( 15737) |
| --- | --- |

| S = 1.051 | Npar= 841 |
| --- | --- |

The following ALERTS were generated. Each ALERT has the format

**test-name_ALERT_alert-type_alert-level**.

Click on the hyperlinks for more details of the test.


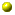
**Alert level C**

[PLAT052_ALERT_1_C](javascript:makeHelpWindow(%22PLAT052.html%22)) Info on Absorption Correction Method Not Given Please Do !

[PLAT220_ALERT_2_C](javascript:makeHelpWindow(%22PLAT220.html%22)) NonSolvent Resd 1 C Ueq(max)/Ueq(min) Range 5.8 Ratio

[PLAT220_ALERT_2_C](javascript:makeHelpWindow(%22PLAT220.html%22)) NonSolvent Resd 1 N Ueq(max)/Ueq(min) Range 5.7 Ratio

[PLAT222_ALERT_3_C](javascript:makeHelpWindow(%22PLAT222.html%22)) NonSolvent Resd 1 H Uiso(max)/Uiso(min) Range 7.7 Ratio

[PLAT242_ALERT_2_C](javascript:makeHelpWindow(%22PLAT242.html%22)) Low 'MainMol' Ueq as Compared to Neighbors of Cu2 Check

[PLAT250_ALERT_2_C](javascript:makeHelpWindow(%22PLAT250.html%22)) Large U3/U1 Ratio for Average U(i,j) Tensor .... 2.7 Note

[PLAT260_ALERT_2_C](javascript:makeHelpWindow(%22PLAT260.html%22)) Large Average Ueq of Residue Including O1_7 0.125 Check

[PLAT260_ALERT_2_C](javascript:makeHelpWindow(%22PLAT260.html%22)) Large Average Ueq of Residue Including O1_4^B 0.120 Check


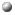
**Alert level G**

[ABSMU01_ALERT_1_G](javascript:makeHelpWindow(%22ABSMU_01.html%22)) Calculation of _exptl_absorpt_correction_mu

not performed for this radiation type.

[PLAT002_ALERT_2_G](javascript:makeHelpWindow(%22PLAT002.html%22)) Number of Distance or Angle Restraints on AtSite 30 Note

[PLAT003_ALERT_2_G](javascript:makeHelpWindow(%22PLAT003.html%22)) Number of Uiso or Uij Restrained non-H Atoms ... 92 Report

[PLAT004_ALERT_5_G](javascript:makeHelpWindow(%22PLAT004.html%22)) Polymeric Structure Found with Maximum Dimension 2 Info

[PLAT007_ALERT_5_G](javascript:makeHelpWindow(%22PLAT007.html%22)) Number of Unrefined Donor-H Atoms .............. 4 Report

[PLAT012_ALERT_1_G](javascript:makeHelpWindow(%22PLAT012.html%22)) N.O.K. _shelx_res_checksum Found in CIF ...... Please Check

[PLAT177_ALERT_4_G](javascript:makeHelpWindow(%22PLAT177.html%22)) The CIF-Embedded .res File Contains DELU Records 1 Report

[PLAT178_ALERT_4_G](javascript:makeHelpWindow(%22PLAT178.html%22)) The CIF-Embedded .res File Contains SIMU Records 1 Report

[PLAT300_ALERT_4_G](javascript:makeHelpWindow(%22PLAT300.html%22)) Atom Site Occupancy of O1_4^A Constrained at 0.5 Check

**And 29 other PLAT300 Alerts**

[PLAT300_ALERT_4_G](javascript:makeHelpWindow(%22PLAT300.html%22)) Atom Site Occupancy of N1_4^A Constrained at 0.5 Check

[PLAT300_ALERT_4_G](javascript:makeHelpWindow(%22PLAT300.html%22)) Atom Site Occupancy of C2_4^A Constrained at 0.5 Check

[PLAT300_ALERT_4_G](javascript:makeHelpWindow(%22PLAT300.html%22)) Atom Site Occupancy of C3_4^A Constrained at 0.5 Check

[PLAT300_ALERT_4_G](javascript:makeHelpWindow(%22PLAT300.html%22)) Atom Site Occupancy of C4_4^A Constrained at 0.5 Check

[PLAT300_ALERT_4_G](javascript:makeHelpWindow(%22PLAT300.html%22)) Atom Site Occupancy of C5_4^A Constrained at 0.5 Check

[PLAT300_ALERT_4_G](javascript:makeHelpWindow(%22PLAT300.html%22)) Atom Site Occupancy of H3A_4^A Constrained at 0.5 Check

[PLAT300_ALERT_4_G](javascript:makeHelpWindow(%22PLAT300.html%22)) Atom Site Occupancy of H3B_4^A Constrained at 0.5 Check

[PLAT300_ALERT_4_G](javascript:makeHelpWindow(%22PLAT300.html%22)) Atom Site Occupancy of H3C_4^A Constrained at 0.5 Check

[PLAT300_ALERT_4_G](javascript:makeHelpWindow(%22PLAT300.html%22)) Atom Site Occupancy of H4A_4^A Constrained at 0.5 Check

[PLAT300_ALERT_4_G](javascript:makeHelpWindow(%22PLAT300.html%22)) Atom Site Occupancy of H4B_4^A Constrained at 0.5 Check

[PLAT300_ALERT_4_G](javascript:makeHelpWindow(%22PLAT300.html%22)) Atom Site Occupancy of H4C_4^A Constrained at 0.5 Check

[PLAT300_ALERT_4_G](javascript:makeHelpWindow(%22PLAT300.html%22)) Atom Site Occupancy of H5A_4^A Constrained at 0.5 Check

[PLAT300_ALERT_4_G](javascript:makeHelpWindow(%22PLAT300.html%22)) Atom Site Occupancy of H5B_4^A Constrained at 0.5 Check

[PLAT300_ALERT_4_G](javascript:makeHelpWindow(%22PLAT300.html%22)) Atom Site Occupancy of H5C_4^A Constrained at 0.5 Check

[PLAT300_ALERT_4_G](javascript:makeHelpWindow(%22PLAT300.html%22)) Atom Site Occupancy of O1_4^B Constrained at 0.5 Check

[PLAT300_ALERT_4_G](javascript:makeHelpWindow(%22PLAT300.html%22)) Atom Site Occupancy of N1_4^B Constrained at 0.5 Check

[PLAT300_ALERT_4_G](javascript:makeHelpWindow(%22PLAT300.html%22)) Atom Site Occupancy of C2_4^B Constrained at 0.5 Check

[PLAT300_ALERT_4_G](javascript:makeHelpWindow(%22PLAT300.html%22)) Atom Site Occupancy of C4_4^B Constrained at 0.5 Check

[PLAT300_ALERT_4_G](javascript:makeHelpWindow(%22PLAT300.html%22)) Atom Site Occupancy of C3_4^B Constrained at 0.5 Check

[PLAT300_ALERT_4_G](javascript:makeHelpWindow(%22PLAT300.html%22)) Atom Site Occupancy of C5_4^B Constrained at 0.5 Check

[PLAT300_ALERT_4_G](javascript:makeHelpWindow(%22PLAT300.html%22)) Atom Site Occupancy of H4D_4^B Constrained at 0.5 Check

[PLAT300_ALERT_4_G](javascript:makeHelpWindow(%22PLAT300.html%22)) Atom Site Occupancy of H4E_4^B Constrained at 0.5 Check

[PLAT300_ALERT_4_G](javascript:makeHelpWindow(%22PLAT300.html%22)) Atom Site Occupancy of H4F_4^B Constrained at 0.5 Check

[PLAT300_ALERT_4_G](javascript:makeHelpWindow(%22PLAT300.html%22)) Atom Site Occupancy of H3D_4^B Constrained at 0.5 Check

[PLAT300_ALERT_4_G](javascript:makeHelpWindow(%22PLAT300.html%22)) Atom Site Occupancy of H3E_4^B Constrained at 0.5 Check

[PLAT300_ALERT_4_G](javascript:makeHelpWindow(%22PLAT300.html%22)) Atom Site Occupancy of H3F_4^B Constrained at 0.5 Check

[PLAT300_ALERT_4_G](javascript:makeHelpWindow(%22PLAT300.html%22)) Atom Site Occupancy of H5D_4^B Constrained at 0.5 Check

[PLAT300_ALERT_4_G](javascript:makeHelpWindow(%22PLAT300.html%22)) Atom Site Occupancy of H5E_4^B Constrained at 0.5 Check

[PLAT300_ALERT_4_G](javascript:makeHelpWindow(%22PLAT300.html%22)) Atom Site Occupancy of H5F_4^B Constrained at 0.5 Check

[PLAT301_ALERT_3_G](javascript:makeHelpWindow(%22PLAT301.html%22)) Main Residue Disorder ..............(Resd 1 ) 9% Note

[PLAT302_ALERT_4_G](javascript:makeHelpWindow(%22PLAT302.html%22)) Anion/Solvent/Minor-Residue Disorder (Resd 3 ) 100% Note

[PLAT302_ALERT_4_G](javascript:makeHelpWindow(%22PLAT302.html%22)) Anion/Solvent/Minor-Residue Disorder (Resd 4 ) 100% Note

[PLAT371_ALERT_2_G](javascript:makeHelpWindow(%22PLAT371.html%22)) Long C(sp2)-C(sp1) Bond C6_1 - C8_1 . 1.43 Ang.

**And 7 other PLAT371 Alerts**

[PLAT371_ALERT_2_G](javascript:makeHelpWindow(%22PLAT371.html%22)) Long C(sp2)-C(sp1) Bond C9_1 - C10_1 . 1.43 Ang.

[PLAT371_ALERT_2_G](javascript:makeHelpWindow(%22PLAT371.html%22)) Long C(sp2)-C(sp1) Bond C12_1 - C16_1 . 1.42 Ang.

[PLAT371_ALERT_2_G](javascript:makeHelpWindow(%22PLAT371.html%22)) Long C(sp2)-C(sp1) Bond C17_1 - C18_1 . 1.45 Ang.

[PLAT371_ALERT_2_G](javascript:makeHelpWindow(%22PLAT371.html%22)) Long C(sp2)-C(sp1) Bond C6_2 - C8_2 . 1.43 Ang.

[PLAT371_ALERT_2_G](javascript:makeHelpWindow(%22PLAT371.html%22)) Long C(sp2)-C(sp1) Bond C9_2 - C10_2 . 1.43 Ang.

[PLAT371_ALERT_2_G](javascript:makeHelpWindow(%22PLAT371.html%22)) Long C(sp2)-C(sp1) Bond C12_2 - C16_2 . 1.42 Ang.

[PLAT371_ALERT_2_G](javascript:makeHelpWindow(%22PLAT371.html%22)) Long C(sp2)-C(sp1) Bond C17_2 - C18_2 . 1.43 Ang.

[PLAT380_ALERT_4_G](javascript:makeHelpWindow(%22PLAT380.html%22)) Incorrectly? Oriented X(sp2)-Methyl Moiety ..... C4_3^B Check

**And 3 other PLAT380 Alerts**

[PLAT380_ALERT_4_G](javascript:makeHelpWindow(%22PLAT380.html%22)) Incorrectly? Oriented X(sp2)-Methyl Moiety ..... C5_3^B Check

[PLAT380_ALERT_4_G](javascript:makeHelpWindow(%22PLAT380.html%22)) Incorrectly? Oriented X(sp2)-Methyl Moiety ..... C4_7 Check

[PLAT380_ALERT_4_G](javascript:makeHelpWindow(%22PLAT380.html%22)) Incorrectly? Oriented X(sp2)-Methyl Moiety ..... C5_7 Check

[PLAT432_ALERT_2_G](javascript:makeHelpWindow(%22PLAT432.html%22)) Short Inter X...Y Contact C4_7 ..C3_4^B 3.16 Ang.

1-x,-1/2+y,1/2-z = 2_645 Check

[PLAT606_ALERT_4_G](javascript:makeHelpWindow(%22PLAT606.html%22)) Solvent Accessible VOID(S) in Structure ........ ! Info

[PLAT720_ALERT_4_G](javascript:makeHelpWindow(%22PLAT720.html%22)) Number of Unusual/Non-Standard Labels .......... 159 Note

[PLAT794_ALERT_5_G](javascript:makeHelpWindow(%22PLAT794.html%22)) Tentative Bond Valency for Cu1 (II) . 2.18 Info

[PLAT860_ALERT_3_G](javascript:makeHelpWindow(%22PLAT860.html%22)) Number of Least-Squares Restraints ............. 752 Note

[PLAT869_ALERT_4_G](javascript:makeHelpWindow(%22PLAT869.html%22)) ALERTS Related to the Use of SQUEEZE Suppressed ! Info

[PLAT883_ALERT_1_G](javascript:makeHelpWindow(%22PLAT883.html%22)) No Info/Value for _atom_sites_solution_primary . Please Do !

[PLAT965_ALERT_2_G](javascript:makeHelpWindow(%22PLAT965.html%22)) The SHELXL WEIGHT Optimisation has not Converged Please Check

# References

Aragao, David, Jun Aishima, Hima Cherukuvada, Robert Clarken, Mark Clift, Nathan Philip Cowieson, Daniel Jesper Ericsson, et al. 2018. “MX2: A High-Flux Undulator Microfocus Beamline Serving Both the Chemical and Macromolecular Crystallography Communities at the Australian Synchrotron.” *Journal of Synchrotron Radiation* 25 (3): 885–91.

Cowieson, Nathan Philip, David Aragao, Mark Clift, Daniel J Ericsson, Christine Gee, Stephen J Harrop, Nathan Mudie, et al. 2015. “MX1: A Bending-Magnet Crystallography Beamline Serving Both Chemical and Macromolecular Crystallography Communities at the Australian Synchrotron.” *Journal of Synchrotron Radiation* 22 (1): 187–90.

García Reyes, Luis Enrique. 2013. *Journal of Chemical Information and Modeling*. Vol. 53.

Hubschle, C B, G M Sheldrick, and B Dittrich. 2011. “ShelXle: A Qt Graphical User Interface for SHELXL.” *Journal of Applied Crystallography* 44 (6): 1281–84. https://doi.org/doi:10.1107/S0021889811043202.

MAYRA, GUALLICHICO. 2013. *Journal of Chemical Information and Modeling* 53 (9): 1689–99.

Spek, Anthony. 2009. “Structure Validation in Chemical Crystallography.” *Acta Crystallographica Section D* 65 (2): 148–55. https://doi.org/doi:10.1107/S090744490804362X.

Spek, Anthony L. 2015. “PLATON SQUEEZE: A Tool for the Calculation of the Disordered Solvent Contribution to the Calculated Structure Factors.” *Acta Crystallographica Section C: Structural Chemistry* 71 (1): 9–18. https://doi.org/10.1107/S2053229614024929.

Thorn, Andrea, Birger Dittrich, and George M Sheldrick. 2012. “Enhanced Rigid-Bond Restraints.” *Acta Crystallographica Section A* 68 (4): 448–51. https://doi.org/doi:10.1107/S0108767312014535.
